# Sound transmission loss of double-walled sandwich cross-ply layered magneto-electro-elastic plates under thermal environment

**DOI:** 10.1038/s41598-022-20965-3

**Published:** 2022-10-05

**Authors:** Nima Refahati, Thira Jearsiripongkul, Chanachai Thongchom, Peyman Roodgar Saffari, Pouyan Roodgar Saffari, Suraparb Keawsawasvong

**Affiliations:** 1 Department of Mechanical Engineering, Faculty of Engineering, Thammasat School of Engineering, Thammasat University, Bangkok, Pathumthani 12120 Thailand; 2 Department of Civil Engineering, Faculty of Engineering, Thammasat School of Engineering, Thammasat University, Bangkok, Pathumthani 12120 Thailand

**Keywords:** Energy science and technology, Engineering, Mathematics and computing

## Abstract

This study offers a comprehensive investigation into the parameters affecting the sound transmission characteristics of a double-walled sandwich magneto-electro-elastic cross-ply layered plate resting on viscoelastic medium in thermal environment. To this end, the walls of this sandwich structure are modeled based on the assumptions of the first-order shear deformation theory. The governing equations are derived via a coupled set of equations targeting vibration and acoustic aspects of the problem after the application of Hamilton’s principle. The obtained equations are then solved by the implementation of double Fourier series and the second velocity potential, giving an accurate estimation of sound transmission loss under initial magnetic and electric potentials, variations of temperature, ply angle, acoustic cavity depth, incident angle of sound waves, and viscoelastic parameters.

## Introduction

Recent years have seen a growing number of studies on the new applications and implementations of devices benefiting from smart material^1–6^. Besides piezoelectric materials, electrorheological and magnetorheological fluids and shape memory alloys, magneto-electro-elastic (MEE) materials have proved to be viable choices thanks to offering the advantages of both composite materials and piezomagnetism/piezoelectricity. Pan et al.^7^ proposed an analytical study to calculate the natural frequencies of multilayered rectangular MEE plates. Vinyas et al.^8^ investigated the free vibration problem of skew MEE plates using higher order shear deformation theory and finite element method (FEM). According to higher order shear deformation theory, Vinyas^9^ studied free vibration carbon nanotube-reinforced MEE skew and rectangular plates. Vinyas et al.^10^ evaluated the natural frequencies of functionally graded (FG) carbon nanotube reinforced MEE plates utilizing higher order shear deformation theory under closed and open electro-magnetic circuit conditions. Based on Golla-Hughes-McTavish technique and geometrically nonlinear vibrations, Esayas et al.^11^ analyzed the influences of porosity and porosity distribution on the nonlinear natural frequencies of FG-MEE plate. Manyo et al.^12^ presented Kelvin–Voigt model to investigate time domains vibration behavior of MEE plates employing dual variable and position (DVP) method.

Such composite materials have been increasingly applied in different areas of technology, especially aerospace and automotive engineering^13,14^. The existence of fibers allows us to refine different conflicting notions such as wear resistance and weight considerations. While there are innumerable geometries for engineering structures, composite laminated plates have gained an excessive amount of attention^15–18^. Zhang et al.^19^ carried out the chaotic dynamic and the chaotic wave motions behaviors of piezoelectric composite laminated plate subjected to the transverse and the in-plane excitations. He et al.^20^ studied the effects of the damping ratio, shape, and frequency of delaminated composite plates and indicated that with increasing the delamination percentage, the modal damping ratio meaningfully increases. Hachemi and Hamza-Cherif^21^ proposed a numerical solution based on hierarchical finite element formulation for investigating the free vibration response of a composite laminated plate. Sharma et al.^22^ carried out the static and dynamic responses of the laminated composite plate with piezoelectric layers employing FEM. Zhang et al.^23^ predicted the dynamic response of rectangular sandwich plates with Fiber metal laminate (FML) composites layers under blast loading for fully clamped boundary conditions. Chen et al.^24^ used Rayleigh–Ritz method and Lagrange multiplier method to obtain the natural frequencies of the trapezoidal bi-stable composite laminate plate. Kiani and Żur^25^ performed the dynamic behavior of graphene platelet reinforced composite skew plates using the first-order shear deformation theory (FSDT) and Halpin–Tsai rule.

Ever since the concept of modern laminated structures came into play, the study on double-walled structures in different areas of mechanical engineering for various applications such as aerospace vehicles increased significantly^26–28^. These structures did not remain limited to the mentioned area and other fields such as acoustics and civil engineering began implementing them^29–32^. One such application is their exceptional noise elimination properties when used correctly, corroborated by a vast array of experimental and analytical studies. The continuous research on their noise cancellation characteristics is one important area where their great potential is being exploited via empirical and analytical means. The literature is rich in studies focusing on the vibroacoustic features of noise transmission of single or double-walled shells and plates^33–40^. Fu et al.^41^ investigated the STL across the stiffened double laminated composite sandwich plate structures under sound wave excitation. Danesh and Ghadami^42^ predicted STL of a rigidly baffled finite rectangular double wall FG piezoelectric plate based on the third-order shear deformation theory (TSDT) and power law model. They indicated that by means of Helium and Hydrogen gases for filling the acoustic cavity between the two piezoelectric plates has a considerable effect on the sound isolation performance. Amirinezhad et al.^43^ analyzed STL across a polymeric foam plate via FSDT. Oliazadeh et al.^44^ experimentally and analytically developed a statistical energy analysis (SEA) model to evaluate the STL of honeycomb sandwich panels.

In spite of various studies on the noise transmission loss characteristics of double-walled plates, there has been no study on the STL of a double-walled sandwich cross-ply layered MEE plate resting on viscoelastic medium and in a thermal environment undergoing harmonic plane sound waves. This study considers the mentioned problem and utilizes the first-order shear deformation theory (FSDT) and Hamilton’s principle to obtain the governing vibroacoustic equations. In addition, the velocity potential technique allows us to solve the equations in view of the properties of the included cavity.

## Preliminary formulations

**Figure 1:**
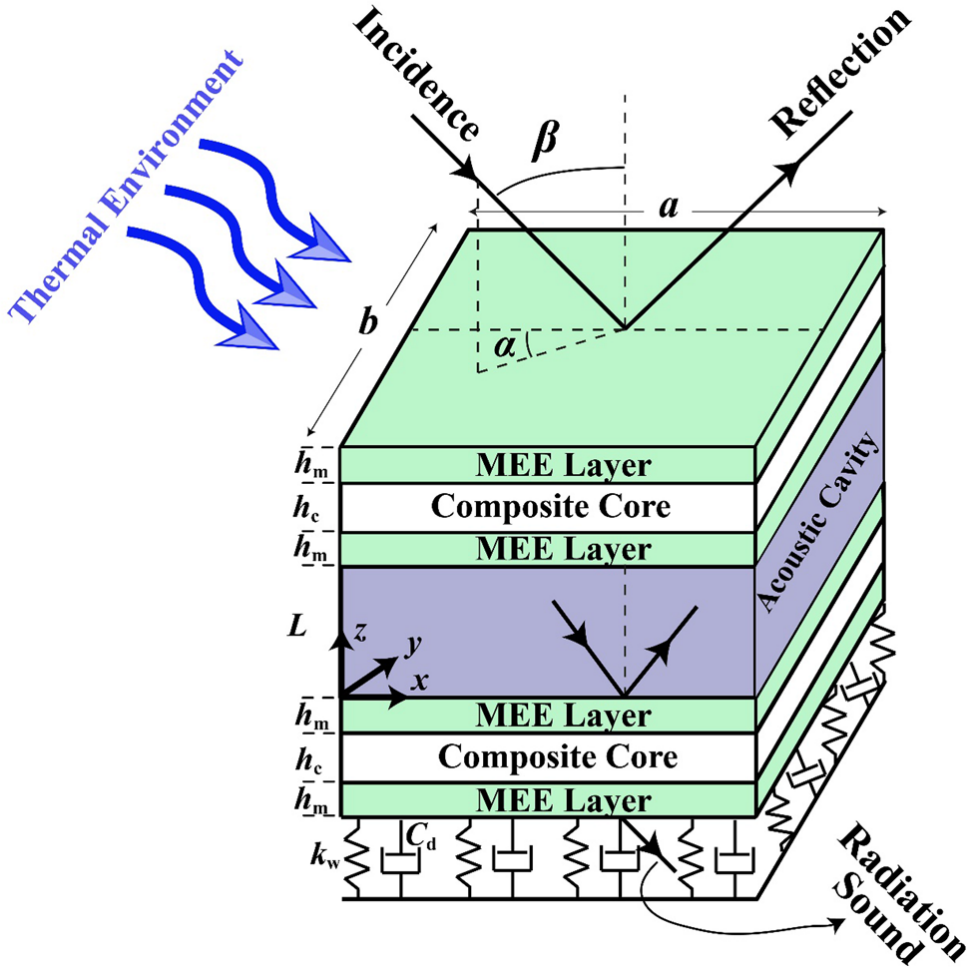
Configuration of double-walled sandwich composite MEE plate resting on viscoelastic medium under incidence wave and thermal environment.

The general assumptions of the problem according to Fig. 1 are discussed as follows. A plane sound wave hits the top side of the double-walled sandwich cross-ply layered MEE plate of the overall dimensions $a\times b$. Also, the double-walled sandwich plate is supposed to be resting on the viscoelastic medium with dashpot coefficient ${c}_{d}$ and transverse stiffness ${k}_{w}$. The azimuth and elevation angles of this time-harmonic sound wave are denoted by $\alpha$ and $\beta$. Furthermore, each side of this structure comprises two similar piezomagnetic layers made from BaTiO_3_-CoFe_2_O_4_ of the thickness ${h}_{m}$ around a cross-ply layered core of the thickness ${h}_{c}$. All MEE plates undergo both electric and magnetic potentials of, respectively, $\Upsilon \left(x,y,z,t\right)$ and $\uppsi \left(x,y,z,t\right)$. Finally, the depth of formed cavity is $L$. Additionally, the system is exposed to a thermal environment with the temperature variation as $\Delta T$.

### Constitutive relations

The underlying equations of motion according to the assumption of FSDT are derived by first considering a displacement field of three components $\left(U,V,W\right)$ for every sandwich MEE plate as in^45^

1 $$\begin{aligned}{U}_{i}\left(x,y,z,t\right)& ={u}_{i}\left(x,y,t\right)+z{\theta }_{xi}\left(x,y,t\right), \\ {V}_{i}\left(x,y,z,t\right)&={v}_{i}\left(x,y,t\right)+z{\theta }_{yi}\left(x,y,t\right), \\ {W}_{i}\left(x,y,z,t\right)&={w}_{i}\left(x,y,t\right), \end{aligned}$$

in which $i=\mathrm{1,2}$, $w$ is the transverse unknown displacement, and for each sandwich composite MEE plate, $v$ and $u$ respectively denote the in-plane deflections of the mid-surface along $y$ and $x$ directions. Additionally, ${\theta }_{x}$ and ${\theta }_{y}$ respectively refer to the rotations of the middle plane along $x$ and $y$ directions. Regarding the linear strain–displacement relation, particular components of the normal strains $\left({\varepsilon }_{xx}, {\varepsilon }_{yy}\right)$ and shear strains $\left({\gamma }_{xz}, {\gamma }_{yz},{\gamma }_{xy}\right)$ are presented as follows

2 $$\begin{aligned} {\varepsilon }_{xxi} &=\frac{{\partial u}_{i}}{\partial x}+z\frac{\partial {\theta }_{xi}}{\partial x}, \;\; {\varepsilon }_{yyi}=\frac{{\partial v}_{i}}{\partial y}+z\frac{\partial {\theta }_{yi}}{\partial y}, {\gamma }_{xzi}={\theta }_{xi}+\frac{\partial {w}_{i}}{\partial x}, \\ {\gamma }_{yzi}&={\theta }_{yi}+\frac{\partial {w}_{i}}{\partial y}, \;\; {\gamma }_{xyi}=\frac{{\partial u}_{i}}{\partial y}+\frac{{\partial v}_{i}}{\partial x}+z\left(\frac{\partial {\theta }_{x}i}{\partial y}+\frac{\partial {\theta }_{yi}}{\partial x}\right),\end{aligned}$$

in which $i=\mathrm{1,2}$. The composite consists of orthotropic layers with different fiber angles. However, every layer must be transferred eccentrically to the coordinates on the axis. The structural stress–strain relationships based on the plane stress mode for cross-ply layered core taking into account the thermal environment are stated as^46^

3 $${\left[\begin{array}{c}{\sigma }_{xxi}\\ {\sigma }_{yyi}\\ {\sigma }_{xyi}\\ {\sigma }_{yzi}\\ {\sigma }_{xzi}\end{array}\right]}_{k}={\left[\begin{array}{ccccc}{\overline{Q} }_{11}& {\overline{Q} }_{12}& 0& 0& 0\\ {\overline{Q} }_{21}& {\overline{Q} }_{22}& 0& 0& 0\\ 0& 0& {\overline{Q} }_{66}& 0& 0\\ 0& 0& 0& {\overline{Q} }_{44}& 0\\ 0& 0& 0& 0& {\overline{Q} }_{55}\end{array}\right]}_{k}{\left[\begin{array}{c}{\varepsilon }_{xxi}\\ {\varepsilon }_{yyi}\\ {\gamma }_{xyi}\\ {\gamma }_{yzi}\\ {\gamma }_{xzi}\end{array}\right]}_{k}-\left[\begin{array}{c}\overline{\alpha }\left(z\right)\\ \overline{\alpha }\left(z\right)\\ 0\\ 0\\ 0\end{array}\right]\Delta T, \quad i=1,2.$$

in which $\overline{\alpha }$ represents the thermal expansion and ${\overline{Q} }_{ij}$ signifies the transformed reduced stiffness coefficients, and for various fiber angles $(\varphi$) are defined as

4 $$\begin{aligned}{\overline{Q} }_{11} & ={Q}_{11}{cos}^{4}\left(\varphi \right)+2\left({Q}_{12}+2{Q}_{66}\right){sin}^{2}\left(\varphi \right){cos}^{2}\left(\varphi \right)+{Q}_{22}{sin}^{4}\left(\varphi \right) \\ {\overline{Q} }_{12}&={\overline{Q} }_{21} =\left({Q}_{11}+{Q}_{22}-4{Q}_{66}\right){sin}^{2}\left(\varphi \right){cos}^{2}\left(\varphi \right)+{Q}_{12}\left({sin}^{4}\left(\varphi \right)+{cos}^{4}\left(\varphi \right)\right), \\{\overline{Q} }_{22}&={Q}_{11}{sin}^{4}\left(\varphi \right)+2\left({Q}_{12}+2{Q}_{66}\right){sin}^{2}\left(\varphi \right){cos}^{2}\left(\varphi \right)+{Q}_{22}{cos}^{4}\left(\varphi \right), \\{\overline{Q} }_{66}&=\left({Q}_{11}+{Q}_{22}-2{Q}_{12}-{Q}_{66}\right){sin}^{2}\left(\varphi \right){cos}^{2}\left(\varphi \right)+{Q}_{66}\left({sin}^{4}\left(\varphi \right)+{sin}^{4}\left(\varphi \right)\right), \\{\overline{Q} }_{44}&={Q}_{44}{cos}^{2}\left(\varphi \right)+{Q}_{55}{sin}^{2}\left(\varphi \right), \\{\overline{Q} }_{55}&={Q}_{55}{cos}^{2}\left(\varphi \right)+{Q}_{44}{sin}^{2}\left(\varphi \right), \\{Q}_{11}&=\frac{{E}_{1}}{1-{\vartheta }_{12}{\vartheta }_{21}}, \; {Q}_{22}=\frac{{E}_{2}}{1-{\vartheta }_{12}{\vartheta }_{21}}, \; {Q}_{12}=\frac{{\vartheta }_{12}{E}_{2}}{1-{\vartheta }_{12}{\vartheta }_{21}}, \; {Q}_{66}={G}_{12},\;{Q}_{44}={G}_{23}, \;{Q}_{55}={G}_{13} \end{aligned}$$

where $i$ can take the values 1 and 2, $E$ is the elastic modulus and $\vartheta$ is the Poisson’s ration. For the sake of completeness, the electric displacement, magnetic induction and stress tensor are described in the form for each MEE plate considering thermal environment^47^

5 $$\begin{aligned} & {\left[\begin{array}{c}\begin{array}{c}{\sigma }_{xxi}\\ {\sigma }_{yyi}\\ {\tau }_{yzi}\end{array}\\ {\tau }_{xzi}\\ {\tau }_{xyi}\end{array}\right]}_{\mathrm{m}}=\left\{\begin{array}{ccccc}{c}_{11}& {c}_{12}& 0& 0& 0\\ {c}_{12}& {c}_{22}& 0& 0& 0\\ 0& 0& {c}_{44}& 0& 0\\ 0& 0& 0& {c}_{55}& 0\\ 0& 0& 0& 0& {c}_{66}\end{array}\right\}\left[\begin{array}{c}\begin{array}{c}{\varepsilon }_{xxi}\\ {\varepsilon }_{yyi}\\ {\gamma }_{yzi}\end{array}\\ {\gamma }_{xzi}\\ {\gamma }_{xyi}\end{array}\right]-\left\{\begin{array}{ccc}0& 0& {e}_{31}\\ 0& 0& {e}_{32}\\ 0& {e}_{24}& 0\\ {e}_{15}& 0& 0\\ 0& 0& 0\end{array}\right\}\left[\begin{array}{c}{\mathrm{\rm E}}_{xi}\\ {\mathrm{\rm E}}_{yi}\\ {\mathrm{\rm E}}_{zi}\end{array}\right]-\left\{\begin{array}{ccc}0& 0& {f}_{31}\\ 0& 0& {f}_{32}\\ 0& {f}_{24}& 0\\ {f}_{15}& 0& 0\\ 0& 0& 0\end{array}\right\}\left[\begin{array}{c}{\mathcal{H}}_{xi}\\ {\mathcal{H}}_{yi}\\ {\mathcal{H}}_{zi}\end{array}\right]-\left[\begin{array}{c}{\lambda }_{1}\\ {\lambda }_{2}\\ 0\\ 0\\ 0\end{array}\right]\Delta T, \\ & \left[\begin{array}{c}{D}_{xi}\\ {D}_{yi}\\ {D}_{zi}\end{array}\right]={\left\{\begin{array}{ccc}0& 0& {e}_{31}\\ 0& 0& {e}_{32}\\ 0& {e}_{24}& 0\\ {e}_{15}& 0& 0\\ 0& 0& 0\end{array}\right\}}^{T}\left[\begin{array}{c}\begin{array}{c}{\varepsilon }_{xxi}\\ {\varepsilon }_{yyi}\\ {\gamma }_{yzi}\end{array}\\ {\gamma }_{xzi}\\ {\gamma }_{xyi}\end{array}\right]+\left\{\begin{array}{ccc}{\kappa }_{11}& 0& 0\\ 0& {\kappa }_{22}& 0\\ 0& 0& {\kappa }_{33}\end{array}\right\}\left[\begin{array}{c}{\mathrm{\rm E}}_{xi}\\ {\mathrm{\rm E}}_{yi}\\ {\mathrm{\rm E}}_{zi}\end{array}\right]+\left\{\begin{array}{ccc}{\mu }_{11}& 0& 0\\ 0& {\mu }_{22}& 0\\ 0& 0& {\mu }_{33}\end{array}\right\}\left[\begin{array}{c}{\mathcal{H}}_{xi}\\ {\mathcal{H}}_{yi}\\ {\mathcal{H}}_{zi}\end{array}\right]+\left[\begin{array}{c}{p}_{1}\\ {p}_{2}\\ {p}_{3}\end{array}\right]\Delta T, \\ & \left[\begin{array}{c}{B}_{xi}\\ {B}_{yi}\\ {B}_{zi}\end{array}\right]={\left\{\begin{array}{ccc}0& 0& {f}_{31}\\ 0& 0& {f}_{32}\\ 0& {f}_{24}& 0\\ {f}_{15}& 0& 0\\ 0& 0& 0\end{array}\right\}}^{T}\left[\begin{array}{c}\begin{array}{c}{\varepsilon }_{xxi}\\ {\varepsilon }_{yyi}\\ {\gamma }_{yzi}\end{array}\\ {\gamma }_{xzi}\\ {\gamma }_{xyi}\end{array}\right]+\left\{\begin{array}{ccc}{\mu }_{11}& 0& 0\\ 0& {\mu }_{22}& 0\\ 0& 0& {\mu }_{33}\end{array}\right\}\left[\begin{array}{c}{\mathrm{\rm E}}_{xi}\\ {\mathrm{\rm E}}_{yi}\\ {\mathrm{\rm E}}_{zi}\end{array}\right]+\left\{\begin{array}{ccc}{\gamma }_{11}& 0& 0\\ 0& {\gamma }_{22}& 0\\ 0& 0& {\gamma }_{33}\end{array}\right\}\left[\begin{array}{c}{\mathcal{H}}_{xi}\\ {\mathcal{H}}_{yi}\\ {\mathcal{H}}_{zi}\end{array}\right]+\left[\begin{array}{c}{q}_{1}\\ {q}_{2}\\ {q}_{3}\end{array}\right]\Delta T, \quad i=\mathrm{1,2} \end{aligned}$$

where terms $\left[\mathbf{D}\right]$ and $\left[\mathbf{B}\right]$ demonstrate the classical components of the electric displacement and magnetic, respectively; $\left[{\varvec{c}}\right], \left[{\varvec{\gamma}}\right],\left[{\varvec{\mu}}\right],\left[{\varvec{\kappa}}\right], \left[{\varvec{f}}\right], \left[{\varvec{e}}\right]$ refer to the components of elasticity tensor, magnetic constants, dielectric, magnetoelectric, piezomagnetic, piezoelectric, respectively. As well, $\left[\mathrm{\rm E}\right]$ and $\left[\mathcal{H}\right]$ present the components of electric and magnetic fields, respectively. However, the Maxwell’s equations need two different assumptions to be satisfied. First, the negative gradient of $\Upsilon (\mathrm{x},\mathrm{y},\mathrm{z},\mathrm{t})$ must be equal to the electric field, and the negative gradient of $\uppsi (\mathrm{x},\mathrm{y},\mathrm{z},\mathrm{t})$ must be equal to the magnetic field. Mathematically, these are expressed as

6 $${\mathrm{\rm E}}_{j}=-\partial\Upsilon /\partial j, {\mathcal{H}}_{j}=-\partial\Psi /\partial j, (j=x,y,z)$$

Considering the boundary condition of the top and bottom sides of MEE layers, one can blend the linear and cosine variations to write the electric potential and magnetic potential in the form^48^

7 $$\begin{aligned} \Upsilon \left(x,y,z,t\right) &=-\mathrm{cos}\left\{\frac{\pi \left[z\pm \left(\frac{{h}_{\mathrm{c}}+{h}_{\mathrm{m}}}{2}\right)\right]}{{h}_{\mathrm{m}}}\right\}\overline{\Upsilon }\left(x,y,t\right)+\frac{2\left[z\pm \left(\frac{{h}_{\mathrm{c}}+{h}_{\mathrm{m}}}{2}\right)\right]V}{{h}_{\mathrm{m}}}, \\ \Psi \left(x,y,z,t\right) &=-\mathrm{cos}\left\{\frac{\pi \left[z\pm \left(\frac{{h}_{\mathrm{c}}+{h}_{\mathrm{m}}}{2}\right)\right]}{{h}_{\mathrm{m}}}\right\}\overline{\Psi }\left(x,y,t\right)+\frac{2\left[z\pm \left(\frac{{h}_{\mathrm{c}}+{h}_{\mathrm{m}}}{2}\right)\right]{\psi }_{0}}{{h}_{\mathrm{m}}}, \end{aligned}$$

Here $\overline{\Upsilon }$ and $\overline{\Psi }$ express the two-dimensional electric and magnetic potentials. Furthermore, $V$ refers to the electric voltage and ${\psi }_{0}$ is the initial magnetic potential.

To obtain the governing equations, Hamilton’s principle is employed as follows

8 $$\underset{{t}_{0}}{\overset{t}{\int }}\left(\updelta {\Pi }_{T}-\updelta {\Pi }_{V}+\updelta {\Pi }_{F}\right)dt=0.$$

where ${\Pi }_{T}$ denotes the virtual kinetic energy; ${\Pi }_{V}$ is the virtual strain energy; ${\Pi }_{F}$ indicates the virtual work done by external applied forces. However, the kinetic energy for double-walled sandwich composite MEE plate is presented as

9 $$\begin{aligned} {\Pi }_{T} & =\sum_{i=1}^{2}{\int }_{{A}_{i}}\left\{{\int }_{-{h}_{\mathrm{m}}-{h}_{c}/2}^{-{h}_{c}/2}{\rho }_{\mathrm{m}}\left[{\left({\dot{U}}_{i}\right)}^{2}+{\left({\dot{V}}_{i}\right)}^{2}+{\left({\dot{W}}_{i}\right)}^{2}\right]dz+{\int }_{-{h}_{c}/2}^{{h}_{c}/2}\rho \left[{\left({\dot{U}}_{i}\right)}^{2}+{\left({\dot{V}}_{i}\right)}^{2}+{\left({\dot{W}}_{i}\right)}^{2}\right]\mathrm{dz} \right. \\ & \quad \left. +{\int }_{-{h}_{c}/2}^{{h}_{\mathrm{m}}+{h}_{c}/2}{\rho }_{\mathrm{m}}\left[{\left({\dot{U}}_{i}\right)}^{2}+{\left({\dot{V}}_{i}\right)}^{2}+{\left({\dot{W}}_{i}\right)}^{2}\right]\mathrm{dz}\right\}\mathrm{d}{A}_{i},\end{aligned}$$

in which $A$ is the cross-sectional area, and ${\rho }_{\mathrm{m}}$ refers to the mass density of MEE layer. The strain energy term is expressed as

10 $$\begin{aligned}{\Pi }_{V} & =\sum_{i=1}^{2}{\int }_{{A}_{i}}\left\{{\int }_{-{h}_{\mathrm{m}}-{h}_{c}/2}^{-{h}_{c}/2}{\left({\sigma }_{xxi}{\varepsilon }_{xxi}+{\sigma }_{yyi}{\varepsilon }_{yyi}+{\tau }_{yzi}{\gamma }_{yzi}+{\tau }_{xzi}{\gamma }_{xzi}+{\tau }_{xyi}{\gamma }_{xyi}\right.}\right.\\&\quad \left.{\left.-{D}_{xi}{\mathrm{\rm E}}_{xi}-{D}_{yi}{\mathrm{\rm E}}_{yi}-{D}_{zi}{\mathrm{\rm E}}_{zi}-{B}_{xi}{\mathcal{H}}_{xi}-{B}_{yi}{\mathcal{H}}_{yi}-{B}_{zi}{\mathcal{H}}_{zi}\vphantom{{\sigma }_{xxi}{\varepsilon }_{xxi}+{\sigma }_{yyi}{\varepsilon }_{yyi}+{\tau }_{yzi}{\gamma }_{yzi}+{\tau }_{xzi}{\gamma }_{xzi}+{\tau }_{xyi}{\gamma }_{xyi}}\right)}_{\mathrm{m}}dz \right. \\ &\quad \left. +{\int }_{-{h}_{c}/2}^{{h}_{c}/2}\left({\sigma }_{xxi}{\varepsilon }_{xxi}+{\sigma }_{yyi}{\varepsilon }_{yyi}+{\tau }_{yzi}{\gamma }_{yzi}+{\tau }_{xzi}{\gamma }_{xzi}+{\tau }_{xyi}{\gamma }_{xyi}\right)\mathrm{d}z\right. \\ &\quad \left.+{\int }_{-{h}_{c}/2}^{{h}_{\mathrm{m}}+{h}_{c}/2}{\left({\sigma }_{xxi}{\varepsilon }_{xxi}+{\sigma }_{yyi}{\varepsilon }_{yyi}+{\tau }_{yzi}{\gamma }_{yzi}+{\tau }_{xzi}{\gamma }_{xzi}+{\tau }_{xyi}{\gamma }_{xyi}-{D}_{xi}{\mathrm{\rm E}}_{xi}\right.}\right.\\&\quad \left.{\left.-{D}_{yi}{\mathrm{\rm E}}_{yi}-{D}_{zi}{\mathrm{\rm E}}_{zi}-{B}_{xi}{\mathcal{H}}_{xi}-{B}_{yi}{\mathcal{H}}_{yi}-{B}_{zi}{\mathcal{H}}_{zi}\right)}_{\mathrm{m}}\mathrm{d}z\vphantom{{\int }_{-{h}_{\mathrm{m}}-{h}_{c}/2}^{-{h}_{c}/2}}\right\}\mathrm{d}{A}_{i}.\end{aligned}$$

The virtual work done by external applied forces due to the thermal environment, viscoelastic medium, incidence sound wave, and initial electric and magnetic potentials is written as

11 $$\begin{aligned}{\Pi }_{F}& ={\int }_{{A}_{1}}\left\{j\omega {\rho }_{0}\left({\Gamma }_{1}-{\Gamma }_{2}\right){w}_{1}+\left({N}_{x}^{E}+{N}_{x}^{M}+{N}_{x}^{T}\right){\left(\frac{\partial {w}_{1}}{\partial x}\right)}^{2}+\left({N}_{y}^{E}+{N}_{y}^{M}+{N}_{y}^{T}\right){\left(\frac{\partial {w}_{1}}{\partial y}\right)}^{2}\right\}\mathrm{d}{A}_{1} \\ & \quad +{\int }_{{A}_{2}}\left\{j\omega {\rho }_{0}\left({\Gamma }_{2}-{\Gamma }_{3}\right){w}_{2}+\left({N}_{x}^{E}+{N}_{x}^{M}+{N}_{x}^{T}\right){\left(\frac{\partial {w}_{2}}{\partial x}\right)}^{2}+\left({N}_{y}^{E}+{N}_{y}^{M}+{N}_{y}^{T}\right){\left(\frac{\partial {w}_{2}}{\partial y}\right)}^{2}+{k}_{W}{w}_{2}+{c}_{d}\frac{\partial {w}_{2}}{\partial t}\right\}\mathrm{d}{A}_{2},\end{aligned}$$

where ${\Gamma }_{1}$, ${\Gamma }_{2}$*,* and ${\Gamma }_{3}$ are, respectively, the velocity potentials in the sound incident area, acoustic cavity, and the transmitted acoustic area. Furthermore, the angular frequency and the air density are displayed, respectively, with $\omega$ and ${\rho }_{0}$. Additionally, $\left({N}_{x}^{T},{N}_{y}^{T}\right), \left({N}_{x}^{E},{N}_{y}^{E}\right)$ and $\left({N}_{x}^{M},{N}_{y}^{M}\right)$ signify the forces due to the thermal variation, initial electric voltage, and initial magnetic potential, along $x$ and $y$ directions, respectively. However, all these loads are expressed as

12 $$\begin{aligned}{N}_{x}^{E}&={\int }_{-{h}_{\mathrm{m}}-{h}_{c}/2}^{-{h}_{c}/2}2{e}_{31}\frac{V}{{h}_{\mathrm{m}}\mathrm{d}z}+{\int }_{\frac{{h}_{c}}{2}}^{{h}_{\mathrm{m}}+\frac{{h}_{c}}{2}}2{e}_{31}\frac{V}{{h}_{\mathrm{m}}\mathrm{d}z}, \\ {N}_{y}^{E}&={\int }_{-{h}_{\mathrm{m}}-{h}_{c}/2}^{-{h}_{c}/2}2{e}_{32}\frac{V}{{h}_{\mathrm{m}}\mathrm{d}z}+{\int }_{\frac{{h}_{c}}{2}}^{{h}_{\mathrm{m}}+\frac{{h}_{c}}{2}}2{e}_{32}\frac{V}{{h}_{\mathrm{m}}\mathrm{d}z}, \\{N}_{x}^{M}&=\frac{{\int }_{-{h}_{\mathrm{m}}-\frac{{h}_{c}}{2}}^{-\frac{{h}_{c}}{2}}2{q}_{31}{\psi }_{0}}{{h}_{\mathrm{m}}\mathrm{d}z}+{\int }_{\frac{{h}_{c}}{2}}^{{h}_{\mathrm{m}}+\frac{{h}_{c}}{2}}2{q}_{31}\frac{{\psi }_{0}}{{h}_{\mathrm{m}}\mathrm{d}z}, \\{N}_{y}^{M}&=\frac{{\int }_{-{h}_{\mathrm{m}}-\frac{{h}_{c}}{2}}^{-\frac{{h}_{c}}{2}}2{q}_{32}{\psi }_{0}}{{h}_{\mathrm{m}}\mathrm{d}z}+{\int }_{\frac{{h}_{c}}{2}}^{{h}_{\mathrm{m}}+\frac{{h}_{c}}{2}}2{q}_{32}\frac{{\psi }_{0}}{{h}_{\mathrm{m}}\mathrm{d}z},\\{N}_{x}^{T}&={\int }_{-{h}_{\mathrm{m}}-h/2}^{-h/2}{\lambda }_{1}\Delta Tdz+{\int }_{-h/2}^{h/2}\overline{\alpha }\Delta Tdz+{\int }_{h/2}^{h/2+{h}_{\mathrm{m}}}{\lambda }_{1}\Delta Tdz, \\{N}_{y}^{T}&={\int }_{-{h}_{\mathrm{m}}-h/2}^{-h/2}{\lambda }_{2}\Delta Tdz+{\int }_{-h/2}^{h/2}\overline{\alpha }\Delta Tdz+{\int }_{h/2}^{h/2+{h}_{\mathrm{m}}}{\lambda }_{2}\Delta Tdz.\end{aligned}$$

It should be noted that in Eq. (11), ${\Gamma }_{1}$, ${\Gamma }_{2}$, and ${\Gamma }_{3}$ respectively denote the velocity potential in the regions 1 to 3 (i.e., the exterior, the cavity, and the sound transmitted region). Now, to find the velocity potential, both positive and negative sound waves are superimposed in all three regions. As a result, one can write^49^

13 $$\begin{aligned}{\Gamma }_{1}\left(x,y,z;t\right)&=I{e}^{-j\left({{k}_{x}x+{k}_{y}y+k}_{z}z-\upomega t\right)}+{T}_{1}{e}^{-j\left({k}_{x}x+{k}_{y}y-{k}_{z}z-\omega t\right)}, \\ {\Gamma }_{2}\left(x,y,z;t\right)&={T}_{2}{e}^{-j\left({{k}_{x}x+{k}_{y}y+k}_{z}z-\omega t\right)}+{T}_{3}{e}^{-j\left({k}_{x}x+{k}_{y}y-{k}_{z}z-\omega t\right)}, \\ {\Gamma }_{3}\left(x,y,z;t\right)&={T}_{4}{e}^{-j\left({k}_{x}x+{k}_{y}y+{k}_{z}z-\omega t\right)}.\end{aligned}$$

where $j=\sqrt{-1}$ and $I$ states the incident sound amplitude. Furthermore, ${T}_{1}, {T}_{2}, {T}_{3}$ and ${T}_{4}$ refers to the unknown modal coefficients of reflected sound wave in the negative-going incident region, positive-going acoustic cavity, negative-going acoustic cavity, and positive-going transmitted wave. Moreover,${k}_{z}={k}_{0}\mathrm{cos}\alpha$,${k}_{y}={k}_{0}\mathrm{sin}\beta \mathrm{sin}\alpha$ and ${k}_{x}={k}_{0}\mathrm{sin}\beta \mathrm{cos}\alpha$, respectively, express the acoustic wavenumbers across z, y, and *x* directions. Additionally, ${k}_{0}=\frac{\upomega }{{c}_{0}}$ is the air acoustic wavenumber and ${c}_{0}$ denotes the air sound velocity.

After substituting Eqs. (9), (10) and (11) into (8) and performing some manipulations, the final form of vibroacoustic equations governing the motion of considered system is written as

14 $$\begin{aligned} & \delta {u}_{i}{:} \; \frac{\partial {N}_{xxi}}{\partial x}+\frac{\partial {N}_{xyi}}{\partial y}={I}_{0}\frac{{\partial }^{2}{u}_{i}}{\partial {t}^{2}}+{I}_{1}\frac{{\partial }^{2}{\theta }_{xi}}{\partial {t}^{2}}, \\ &\delta {v}_{i}{:} \; \frac{\partial {N}_{xyi}}{\partial x}+\frac{\partial {N}_{yyi}}{\partial y}={I}_{0}\frac{{\partial }^{2}{v}_{i}}{\partial {t}^{2}}+{I}_{1}\frac{{\partial }^{2}{\theta }_{yi}}{\partial {t}^{2}}, \\ &\delta {w}_{i}{:} \; \frac{\partial {Q}_{xxi}}{\partial x}+\frac{\partial {Q}_{yyi}}{\partial y}+\left({N}_{x}^{E}+{N}_{x}^{M}+{N}_{x}^{T}\right)\frac{{\partial }^{2}{w}_{i}}{\partial {x}^{2}}\\ & \qquad+\left({N}_{y}^{E}+{N}_{y}^{M}+{N}_{y}^{T}\right)\frac{{\partial }^{2}{w}_{i}}{\partial {y}^{2}}+{k}_{W}{w}_{2}+{c}_{d}\frac{\partial {w}_{2}}{\partial t}={I}_{0}\frac{{\partial }^{2}{w}_{i}}{\partial {t}^{2}}+{q}_{i} \\ & \delta {\theta }_{xi}{:} \; \frac{\partial {M}_{xxi}}{\partial x}+\frac{\partial {M}_{xyi}}{\partial y}-{Q}_{xzi}={I}_{1}\frac{{\partial }^{2}{u}_{i}}{\partial {t}^{2}}+{I}_{2}\frac{{\partial }^{2}{\theta }_{xi}}{\partial {t}^{2}}, \\& \delta {\theta }_{yi}{:} \; \frac{\partial {M}_{yyi}}{\partial y}+\frac{\partial {M}_{xyi}}{\partial x}-{Q}_{yzi}={I}_{1}\frac{{\partial }^{2}{v}_{i}}{\partial {t}^{2}}+{I}_{2}\frac{{\partial }^{2}{\theta }_{yi}}{\partial {t}^{2}}, \\ & \delta {\overline{\Upsilon } }_{i}{:} \; {\int }_{-{h}_{\mathrm{m}}-{h}_{c}/2}^{-{h}_{c}/2}\left(\frac{\partial {D}_{xi}}{\partial x}\mathrm{cos}\left\{\frac{\pi \left[z+\left(\frac{{h}_{\mathrm{c}}+{h}_{\mathrm{m}}}{2}\right)\right]}{{h}_{\mathrm{m}}}\right\}+\frac{\partial {D}_{yi}}{\partial y}\mathrm{cos}\left\{\frac{\pi \left[z+\left(\frac{{h}_{\mathrm{c}}+{h}_{\mathrm{m}}}{2}\right)\right]}{{h}_{\mathrm{m}}}\right\}+\frac{\pi }{{h}_{\mathrm{m}}}{D}_{zi}\mathrm{sin}\left\{\frac{\pi \left[z+\left(\frac{{h}_{\mathrm{c}}+{h}_{\mathrm{m}}}{2}\right)\right]}{{h}_{\mathrm{m}}}\right\}\right)\mathrm{d}z \\&\quad +{\int }_{{h}_{c}/2}^{{h}_{\mathrm{m}}+{h}_{c}/2}\left(\frac{\partial {D}_{xi}}{\partial x}\mathrm{cos}\left\{\frac{\pi \left[z-\left(\frac{{h}_{\mathrm{c}}+{h}_{\mathrm{m}}}{2}\right)\right]}{{h}_{\mathrm{m}}}\right\}+\frac{\partial {D}_{yi}}{\partial y}\mathrm{cos}\left\{\frac{\pi \left[z-\left(\frac{{h}_{\mathrm{c}}+{h}_{\mathrm{m}}}{2}\right)\right]}{{h}_{\mathrm{m}}}\right\}+\frac{\pi }{{h}_{\mathrm{m}}}{D}_{zi}\mathrm{sin}\left\{\frac{\pi \left[z-\left(\frac{{h}_{\mathrm{c}}+{h}_{\mathrm{m}}}{2}\right)\right]}{{h}_{\mathrm{m}}}\right\}\right)=0, \\ & \delta {\overline{\Psi } }_{i}{:} \; {\int }_{-{h}_{\mathrm{m}}-{h}_{c}/2}^{-{h}_{c}/2}\left(\frac{\partial {B}_{xi}}{\partial x}\mathrm{cos}\left\{\frac{\pi \left[z+\left(\frac{{h}_{\mathrm{c}}+{h}_{\mathrm{m}}}{2}\right)\right]}{{h}_{\mathrm{m}}}\right\}+\frac{\partial {B}_{yi}}{\partial y}\mathrm{cos}\left\{\frac{\pi \left[z+\left(\frac{{h}_{\mathrm{c}}+{h}_{\mathrm{m}}}{2}\right)\right]}{{h}_{\mathrm{m}}}\right\}+\frac{\pi }{{h}_{\mathrm{m}}}{B}_{zi}\mathrm{sin}\left\{\frac{\pi \left[z+\left(\frac{{h}_{\mathrm{c}}+{h}_{\mathrm{m}}}{2}\right)\right]}{{h}_{\mathrm{m}}}\right\}\right)\mathrm{d}z\\&\quad +{\int }_{{h}_{c}/2}^{{h}_{\mathrm{m}}+{h}_{c}/2}\left(\frac{\partial {B}_{xi}}{\partial x}\mathrm{cos}\left\{\frac{\pi \left[z-\left(\frac{{h}_{\mathrm{c}}+{h}_{\mathrm{m}}}{2}\right)\right]}{{h}_{\mathrm{m}}}\right\}+\frac{\partial {B}_{yi}}{\partial y}\mathrm{cos}\left\{\frac{\pi \left[z-\left(\frac{{h}_{\mathrm{c}}+{h}_{\mathrm{m}}}{2}\right)\right]}{{h}_{\mathrm{m}}}\right\}+\frac{\pi }{{h}_{\mathrm{m}}}{B}_{zi}\mathrm{sin}\left\{\frac{\pi \left[z-\left(\frac{{h}_{\mathrm{c}}+{h}_{\mathrm{m}}}{2}\right)\right]}{{h}_{\mathrm{m}}}\right\}\right)=0, \end{aligned}$$

in which $i=\mathrm{1,2}$. Furthermore, ${q}_{1}=j\omega {\rho }_{0}\left({\Gamma }_{1}-{\Gamma }_{2}\right), {q}_{2}=j\omega {\rho }_{0}\left({\Gamma }_{2}-{\Gamma }_{3}\right)$. Besides, the forces, bending and shear moments ${N}_{xx}, {N}_{xy}, {N}_{yy},{M}_{xx}, {M}_{xy}, {M}_{yy},{Q}_{xz}, {Q}_{yz}$ and mass inertia terms ${I}_{0},{I}_{1},{I}_{2}$ are written as

15 $$\begin{aligned}\left\{{N}_{xxi}, {N}_{yyi},{N}_{xyi} \right\} & ={\int }_{-{h}_{\mathrm{m}}-{h}_{c}/2}^{-{h}_{c}/2}{\left\{{\sigma }_{xxi},{\sigma }_{yyi}, {\tau }_{xyi}\right\}}_{\mathrm{m}}dz +{\int }_{-{h}_{c}/2}^{{h}_{c}/2}\left\{{\sigma }_{xxi},{\sigma }_{yyi}, {\tau }_{xyi}\right\}dz \\ & \quad + {\int }_{{h}_{c}/2}^{{h}_{c}/2+{h}_{\mathrm{m}}}{\left\{{\sigma }_{xxi},{\sigma }_{yyi}, {\tau }_{xyi}\right\}}_{\mathrm{m}}dz , \\ \left\{{M}_{xxi}, {M}_{yyi},{M}_{xyi} \right\}&={\int }_{-{h}_{\mathrm{m}}-{h}_{c}/2}^{-{h}_{c}/2}{\left\{{\sigma }_{xxi},{\sigma }_{yyi}, {\tau }_{xyi}\right\}}_{\mathrm{m}}zdz+{\int }_{-{h}_{c}/2}^{{h}_{c}/2}\left\{{\sigma }_{xxi},{\sigma }_{yyi}, {\tau }_{xyi}\right\}zdz \\ & \quad +{\int }_{{h}_{c}/2}^{{h}_{c}/2+{h}_{\mathrm{m}}}{\left\{{\sigma }_{xxi},{\sigma }_{yyi}, {\tau }_{xyi}\right\}}_{\mathrm{m}}zdz, \\ \left\{{Q}_{xzi}, {Q}_{yzi} \right\} & ={k}_{s}\left[{\int }_{-{h}_{\mathrm{m}}-{h}_{c}/2}^{-{h}_{c}/2}{\left\{{\tau }_{xzi},{\tau }_{yzi}\right\}}_{\mathrm{m}}dz+{\int }_{-{h}_{c}/2}^{{h}_{c}/2}\left\{{\tau }_{xzi},{\tau }_{yzi}\right\}dz +{\int }_{{h}_{c}/2}^{{h}_{c}/2+{h}_{\mathrm{m}}}{\left\{{\tau }_{xzi},{\tau }_{yzi}\right\}}_{\mathrm{m}}dz\right], \\ {I}_{0}&={\int }_{-{h}_{\mathrm{m}}-{h}_{c}/2}^{-{h}_{c}/2}{\rho }_{\mathrm{m}}dz+\sum_{0}^{N}{\rho }^{k}\left({h}_{k}-{h}_{k-1}\right)+{\int }_{{h}_{c}/2}^{{h}_{c}/2+{h}_{\mathrm{m}}}{\rho }_{\mathrm{m}}dz, \\ {I}_{1}& ={\int }_{-{h}_{\mathrm{m}}-{h}_{c}/2}^{-{h}_{c}/2}{\rho }_{\mathrm{m}}zdz+\frac{1}{2}\sum_{0}^{N}{\rho }^{k}\left({{h}_{k}}^{2}-{{h}_{k-1}}^{2}\right)+{\int }_{{h}_{c}/2}^{{h}_{c}/2+{h}_{\mathrm{m}}}{\rho }_{\mathrm{m}}zdz \\ {I}_{1}&={\int }_{-{h}_{\mathrm{m}}-{h}_{c}/2}^{-{h}_{c}/2}{\rho }_{\mathrm{m}}{z}^{2}dz+\frac{1}{3}\sum_{0}^{N}{\rho }^{k}\left({{h}_{k}}^{3}-{{h}_{k-1}}^{3}\right)+{\int }_{{h}_{c}/2}^{{h}_{c}/2+{h}_{\mathrm{m}}}{\rho }_{\mathrm{m}}{z}^{2}dz\end{aligned}$$

Here $i=\mathrm{1,2}$. Also, ${k}_{s}=5/6$ signifies the shear correction term.

Lastly, replacing Eq. (15) into Eq. (14), the governing vibroacoustic equations in terms of the double-walled sandwich plate displacement are obtained and provided in Appendix A. It is supposed that the electric and magnetic potentials at the ends of each MEE plate is zero, and the simply supported boundary conditions are considered for all four edges of each sandwich composite MEE plates

16 $$\begin{aligned} {u}_{i}\left(x,0,t\right)& ={u}_{i}\left(x,b,t\right)={v}_{i}\left(0,y,t\right)={v}_{i}\left(a,y,t\right)=0, \\ {w}_{i}\left(x,0,t\right)& ={w}_{i}\left(x,b,t\right)={w}_{i}\left(0,y,t\right)={w}_{i}\left(a,y,t\right)=0, \\ {\theta }_{xi}\left(x,0,t\right)& ={\theta }_{xi}\left(x,b,t\right)={\theta }_{yi}\left(0,y,t\right)={\theta }_{yi}\left(a,y,t\right)=0, \\ {\overline{\Upsilon } }_{i}\left(x,0,\tau \right)& ={\overline{\Upsilon } }_{i}\left(x,b,t\right)={\overline{\Psi } }_{i}\left(0,y,t\right)={\overline{\Psi } }_{i}\left(a,y,t\right)=0, \\ {\overline{\Upsilon } }_{i}\left(0,y,\tau \right)& ={\overline{\Upsilon } }_{i}\left(a,y,t\right)={\overline{\Psi } }_{i}\left(x,0,t\right)={\overline{\Psi } }_{i}\left(x,b,t\right)=0, \\ {M}_{xxi}\left(0,y,t\right)& ={M}_{xxi}\left(a,y,t\right)={M}_{yyi}\left(x,0,t\right)={M}_{yyi}\left(x,b,t\right)=0, \quad i=\mathrm{1,2}. \end{aligned}$$

The following suitable expressions are used for the associated boundary conditions as

17 $$\begin{aligned}\left\{{u}_{i},{\theta }_{xi} \right\}&=\sum_{m=1}^{\infty }\sum_{n=1}^{\infty }\mathrm{cos}\left(m\pi x/a\right)\mathrm{sin}\left(n\pi y/b\right)\left\{{\widetilde{u}}_{i},{\widetilde{\theta }}_{xi}\right\}{e}^{j\omega t}, \\ \left\{{v}_{i},{\theta }_{yi} \right\} &=\sum_{m=1}^{\infty }\sum_{n=1}^{\infty }\mathrm{sin}\left(m\pi x/a\right)\mathrm{cos}\left(n\pi y/b\right)\left\{{\widetilde{v}}_{i},{\widetilde{\theta }}_{yi}\right\}{e}^{j\omega t}, \\ \left\{{w}_{i},{\overline{\Upsilon } }_{i},{\overline{\Psi } }_{i}\right\}&=\sum_{m=1}^{\infty }\sum_{n=1}^{\infty }\mathrm{sin}\left(m\pi x/a\right)\mathrm{sin}\left(n\pi y/b\right)\left\{{\widetilde{w}}_{i},{\widetilde{\Upsilon }}_{i},{\widetilde{\Psi }}_{i}\right\}{e}^{j\omega t}, \quad i=1,2\end{aligned}$$

in which ${\widetilde{u}}_{i},{\widetilde{\theta }}_{xi}, {\widetilde{v}}_{i},{\widetilde{\theta }}_{yi},{\widetilde{w}}_{i},{\widetilde{\Upsilon }}_{i},{\widetilde{\Psi }}_{i}$ are the unknown modal coefficients. Furthermore, $m$ and $n$ express the half wave numbers along *x* and *y* directions, respectively.

### Acoustic model

Now, the velocity potential in all three regions can be completely defined with the aid of the transverse modal function ${A}_{mn}=\mathrm{sin}\left(m\pi x/a \right)\mathrm{sin}\left(n\pi y/b\right)$ in view of Eq. (13) as in^49^

18 $$\begin{aligned}{\Gamma }_{1}\left(x,y,z;t\right)& =\sum_{m=1}^{\infty }\sum_{n=1}^{\infty }{I}_{mn}{A}_{mn}\left(x,y\right){e}^{-j\left({k}_{z}z-\omega t\right)}+\sum_{m=1}^{\infty }\sum_{n=1}^{\infty }{T}_{1}{A}_{mn}\left(x,y\right){e}^{-j\left(-{k}_{z}z-\omega t\right)} \\ {\Gamma }_{1}\left(x,y,z;t\right) & =\sum_{m=1}^{\infty }\sum_{n=1}^{\infty }{T}_{2}{A}_{mn}\left(x,y\right){e}^{-j\left({k}_{z}z-\omega t\right)}+\sum_{m=1}^{\infty }\sum_{n=1}^{\infty }{T}_{3}{A}_{mn}\left(x,y\right){e}^{-j\left(-{k}_{z}z-\omega t\right)}, \\ {\Gamma }_{1}\left(x,y,z;t\right)& =\sum_{m=1}^{\infty }\sum_{n=1}^{\infty }{T}_{4}{A}_{mn}\left(x,y\right){e}^{-j\left({k}_{z}z-\omega t\right)} \end {aligned}$$

The orthogonality property of modal equations must be used to describe the modal amplitude of the plate that corresponds to the incoming sound wave. For this special case, ${I}_{mn}=4\left({I}_{0}/ab\right){\int }_{0}^{a}{\int }_{0}^{b}{e}^{-j\left({k}_{x}x+{k}_{y}y\right)}\mathrm{sin}(m\pi x/a)\mathrm{sin}(n\pi y/b)\mathrm{d}y\mathrm{d}x$, where ${I}_{0}$ is the incident wave amplitude. At this point, one should find ${T}_{1}$ to ${T}_{4}$. To this aim, the normal velocity at the shared boundary of fluid and structure for each sandwich composite MEE plate should be equal as in

19 $$\begin{aligned} & -\frac{\partial {\Gamma }_{1}}{\partial z}\left|\begin{array}{c}\\ z=L+2{h}_{\mathrm{m}}+{h}_{\mathrm{c}}\end{array}\right.=j\omega { w}_{1}, \;\;- \frac{\partial {\Gamma }_{2}}{\partial z}\left|\begin{array}{c}\\ z=L\end{array}\right.=j\omega {w}_{1}, \\ & -\frac{\partial {\Gamma }_{2}}{\partial z}\left|\begin{array}{c}\\ z=0\end{array}\right.=j\omega {w}_{2},\;\; -\frac{\partial {\Gamma }_{3}}{\partial z}\left|\begin{array}{c}\\ z=-\left(2{h}_{\mathrm{m}}+{h}_{\mathrm{c}}\right)\end{array}\right.=j\omega {w}_{2}.\end{aligned}$$

All of the unknown modal coefficients ${T}_{1}$ to ${T}_{4}$ can be obtained by replacing Eqs. (17) and (18) in Eq. (19) as

20 $$\begin{aligned} & {T}_{1}={I}_{mn}{e}^{-2j{k}_{z}\left(L+2{h}_{\mathrm{m}}+{h}_{\mathrm{c}}\right)}-\omega \frac{{\widetilde{w}}_{1}{e}^{-j{k}_{z}\left(L+2{h}_{\mathrm{m}}+{h}_{\mathrm{c}}\right)}}{{k}_{z,air}},{T}_{2}=\omega \frac{\left({\widetilde{w}}_{1}-{\widetilde{w}}_{2}{e}^{j{k}_{z}L}\right)}{{k}_{z}\left({e}^{-j{k}_{z}L}-{e}^{j{k}_{z}L}\right)} \\ & {T}_{3}=\omega \frac{\left({\widetilde{w}}_{1}-{\widetilde{w}}_{2}{e}^{-j{k}_{z}L}\right)}{{k}_{z}\left({e}^{-j{k}_{z}L}-{e}^{j{k}_{z}L}\right)}, \;\; {T}_{4}=\omega \frac{{\widetilde{w}}_{2}{e}^{-j{k}_{z}\left(2{h}_{\mathrm{m}}+{h}_{\mathrm{c}}\right)}}{{k}_{z}}. \end{aligned}$$

Substituting Eqs. (17) and (18) in the previously-obtained governing equations, i.e., equations (A1)–(A7), leads to the equilibrium equations in a $14\times 14$ matrix format:

21 $$\left[\begin{array}{cccccccccccccc}{\kappa }_{\mathrm{1,1}}& {\kappa }_{\mathrm{1,2}}& 0& {\kappa }_{\mathrm{1,4}}& {\kappa }_{\mathrm{1,5}}& {\kappa }_{\mathrm{1,6}}& {\kappa }_{1,7}& 0& 0& 0& 0& 0& 0& 0\\ {\kappa }_{\mathrm{2,1}}& {\kappa }_{\mathrm{2,2}}& 0& {\kappa }_{\mathrm{2,4}}& {\kappa }_{\mathrm{2,5}}& {\kappa }_{\mathrm{2,6}}& {\kappa }_{\mathrm{2,7}}& 0& 0& 0& 0& 0& 0& 0\\ 0& 0& 0& {\kappa }_{\mathrm{3,4}}& {\kappa }_{\mathrm{3,5}}& {\kappa }_{\mathrm{3,6}}& {\kappa }_{\mathrm{3,7}}& 0& 0& {\kappa }_{\mathrm{3,10}}& 0& 0& 0& 0\\ {\kappa }_{\mathrm{4,1}}& {\kappa }_{\mathrm{4,2}}& {\kappa }_{\mathrm{4,3}}& {\kappa }_{\mathrm{4,4}}& {\kappa }_{\mathrm{4,5}}& {\kappa }_{\mathrm{4,6}}& {\kappa }_{\mathrm{4,7}}& 0& 0& 0& 0& 0& 0& 0\\ {\kappa }_{\mathrm{5,1}}& {\kappa }_{\mathrm{5,2}}& {\kappa }_{\mathrm{5,3}}& {\kappa }_{\mathrm{5,4}}& {\kappa }_{\mathrm{5,5}}& {\kappa }_{\mathrm{5,6}}& {\kappa }_{\mathrm{5,7}}& 0& 0& 0& 0& 0& 0& 0\\ {\kappa }_{\mathrm{6,1}}& {\kappa }_{\mathrm{6,2}}& {\kappa }_{\mathrm{6,3}}& {\kappa }_{\mathrm{6,4}}& {\kappa }_{\mathrm{6,5}}& {\kappa }_{\mathrm{6,6}}& {\kappa }_{\mathrm{6,7}}& 0& 0& 0& 0& 0& 0& 0\\ {\kappa }_{\mathrm{7,1}}& {\kappa }_{\mathrm{7,2}}& {\kappa }_{\mathrm{7,3}}& {\kappa }_{\mathrm{7,4}}& {\kappa }_{\mathrm{7,5}}& {\kappa }_{\mathrm{7,6}}& {\kappa }_{\mathrm{7,7}}& 0& 0& 0& 0& 0& 0& 0\\ 0& 0& 0& 0& 0& 0& 0& {\kappa }_{\mathrm{8,8}}& {\kappa }_{\mathrm{8,9}}& 0& {\kappa }_{\mathrm{8,11}}& {\kappa }_{\mathrm{8,12}}& {\kappa }_{\mathrm{8,13}}& {\kappa }_{\mathrm{8,14}}\\ 0& 0& 0& 0& 0& 0& 0& {\kappa }_{\mathrm{9,8}}& {\kappa }_{\mathrm{9,9}}& 0& {\kappa }_{\mathrm{9,11}}& {\kappa }_{\mathrm{9,12}}& {\kappa }_{\mathrm{9,13}}& {\kappa }_{\mathrm{9,14}}\\ 0& 0& {\kappa }_{\mathrm{10,3}}& 0& 0& 0& 0& 0& 0& {\kappa }_{\mathrm{10,10}}& {\kappa }_{\mathrm{10,11}}& {\kappa }_{\mathrm{10,12}}& {\kappa }_{\mathrm{10,13}}& {\kappa }_{\mathrm{10,14}}\\ 0& 0& 0& 0& 0& 0& 0& {\kappa }_{\mathrm{11,8}}& {\kappa }_{\mathrm{11,9}}& {\kappa }_{\mathrm{11,10}}& {\kappa }_{\mathrm{11,11}}& {\kappa }_{\mathrm{11,12}}& {\kappa }_{\mathrm{11,13}}& {\kappa }_{\mathrm{11,14}}\\ 0& 0& 0& 0& 0& 0& 0& {\kappa }_{\mathrm{12,8}}& {\kappa }_{\mathrm{12,9}}& {\kappa }_{\mathrm{12,10}}& {\kappa }_{\mathrm{12,11}}& {\kappa }_{\mathrm{12,12}}& {\kappa }_{\mathrm{12,13}}& {\kappa }_{\mathrm{12,14}}\\ 0& 0& 0& 0& 0& 0& 0& {\kappa }_{\mathrm{13,8}}& {\kappa }_{\mathrm{13,9}}& {\kappa }_{\mathrm{13,10}}& {\kappa }_{\mathrm{13,11}}& {\kappa }_{\mathrm{13,12}}& {\kappa }_{\mathrm{13,13}}& {\kappa }_{\mathrm{13,14}}\\ 0& 0& 0& 0& 0& 0& 0& {\kappa }_{\mathrm{14,8}}& {\kappa }_{\mathrm{14,9}}& {\kappa }_{\mathrm{14,10}}& {\kappa }_{\mathrm{14,11}}& {\kappa }_{\mathrm{14,12}}& {\kappa }_{\mathrm{14,13}}& {\kappa }_{\mathrm{14,14}}\end{array}\right]\left[\begin{array}{c}{\widetilde{u}}_{1}\\ {\widetilde{v}}_{1}\\ \begin{array}{c}\begin{array}{c}\begin{array}{c}\begin{array}{c}{\widetilde{w}}_{1}\\ {\widetilde{\theta }}_{x1}\\ {\widetilde{\theta }}_{y1}\\ {\widetilde{\Upsilon }}_{1}\end{array}\\ {\widetilde{\Psi }}_{1}\\ {\widetilde{u}}_{2}\end{array}\\ {\widetilde{v}}_{2}\\ {\widetilde{w}}_{2}\end{array}\\ {\widetilde{\theta }}_{x2}\\ {\widetilde{\theta }}_{y2}\end{array}\\ {\widetilde{\Upsilon }}_{2}\\ {\widetilde{\Psi }}_{2}\end{array}\right]=\left[\begin{array}{c}\begin{array}{c}\begin{array}{c}\begin{array}{c}\begin{array}{c}0\\ 0\\ F\\ 0\end{array}\\ 0\\ 0\\ 0\\ 0\end{array}\\ 0\\ 0\end{array}\\ 0\\ 0\end{array}\\ 0\\ 0\end{array}\right]$$

in which ${\kappa }_{i,j}$ and $F$ are stated in Appendix B.

### STL relation

Finally, the sound transmission loss can be obtained by inversing the value of power transmission coefficient. STL is primarily expressed in decibels (dB). For the problem at hand, this is written as^50^

22 $$STL=10{log}_{10}\left(\frac{\sum_{m=1}^{\infty }\sum_{n=1}^{\infty }{\left|{I}_{mn}+{T}_{1}\right|}^{2}}{\sum_{m=1}^{\infty }\sum_{n=1}^{\infty }{\left|{T}_{4}\right|}^{2}}\right),$$

## Results and discussion

Before offering the findings, some simplified cases are evaluated to show the accuracy of the developed method. The parameters used (except those in the verification section) are displayed in Table 1^51,52^.

**Table 1:** Material characteristics of sandwich cross-ply layered MEE plate and acoustic medium.

Properties (MEE layer)	${\mathrm{BaTiO}}_{3}-{\mathrm{CoFe}}_{2}{\mathrm{O}}_{4}$
Elastic (GPa)	${c}_{11}=226, {c}_{12}=125, {c}_{22}=226,$ ${c}_{44}=44.2, {c}_{55}=44.2, {c}_{66}=51$
Piezoelectric ($\mathrm{C }{\mathrm{m}}^{-2}$)	${e}_{31}=-2.2, {e}_{32}=-2.2,$ ${e}_{24}=5.8, {e}_{15}=5.8$
Dielectric (${10}^{-9}\mathrm{C }{\mathrm{V}}^{-1} {\mathrm{m}}^{-1}$)	${\kappa }_{11}=5.64, {\kappa }_{22}=5.64,$ ${\kappa }_{33}=6.35$
Piezomagnetic ($\mathrm{N} {\mathrm{A}}^{-1} {\mathrm{m}}^{-1}$)	${f}_{31}=290.1, {e}_{32}=290.1,$ ${e}_{24}=275, {e}_{15}=275$
Magnetoelectric (${10}^{-12}\mathrm{N S }{\mathrm{V}}^{-1} {\mathrm{C}}^{-1}$)	${\mu }_{11}=5.367, {\mu }_{11}=5.367,$ ${\mu }_{33}=2737.5$
Magnetic (${10}^{-6}\mathrm{N }{\mathrm{s}}^{2} {\mathrm{C}}^{-2}$)	${\gamma }_{11}=-297, {\gamma }_{22}=-297,$ ${\gamma }_{33}=83.5$
Mass density ($\mathrm{kg}$ ${\mathrm{m}}^{-3}$)	${\rho }_{\mathrm{m}}=5550$
Thermal moduli (${10}^{-5}\mathrm{N}/\mathrm{K}{\mathrm{ m}}^{2}$)	${\lambda }_{1}={\lambda }_{2}=4.74$
Properties (composite core)	Graphite/epoxy
Elastic (GPa)	${\mathrm{E}}_{1}=181, {\mathrm{E}}_{2}=103, {\mathrm{G}}_{12}=7.17, {\mathrm{G}}_{13}=7.17, {\mathrm{G}}_{23}=2.87$
Poisson’s ratio	${\mathrm{\vartheta }}_{12}=0.28$
Mass density ($\mathrm{kg}$ ${\mathrm{m}}^{-3}$)	$\rho =1580$
Properties (acoustic medium)	Air
Sound speed ($\mathrm{m} {\mathrm{s}}^{-1}$)	${c}_{0}=343$
Mass density ($\mathrm{kg}$ ${\mathrm{m}}^{-3}$)	${\rho }_{0}=1.21$

### Convergence checking

**Figure 2:**
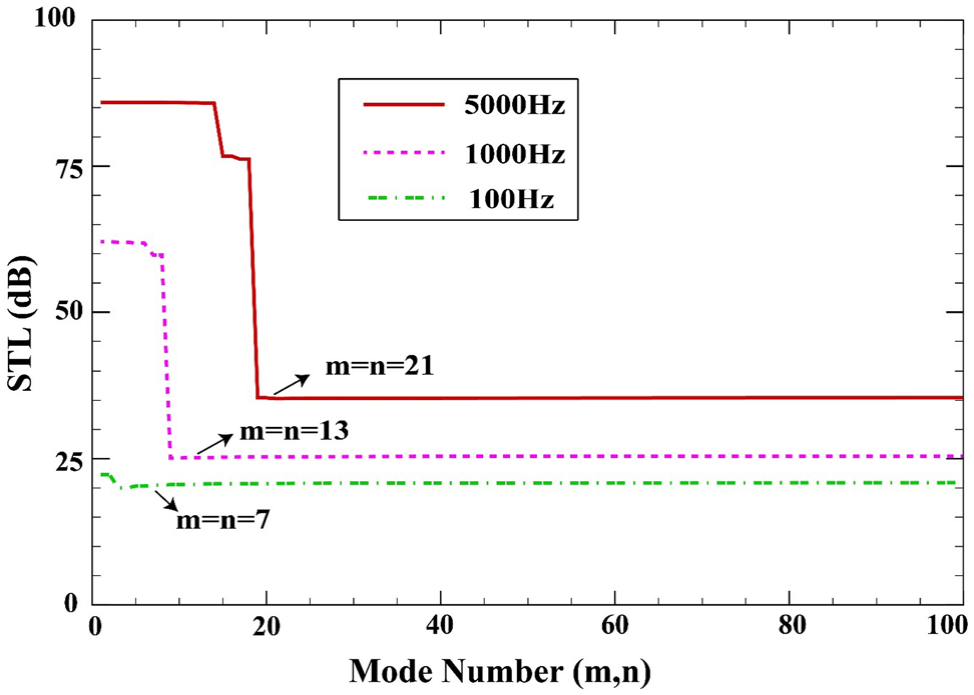
Mode convergence against the truncation numbers $m$ and $n$.

The use of double Fourier series imposes the need for a sufficient number of modes so that the convergence is achieved. As a simple yet effective method, a classic trial-and-error scheme is employed here where the parameters $m$ and $n$ are successively increased and the stability of the numerical value of the response is observed. For a sample case as in Fig. 2, one observes that 441 terms ($m, n = 21$) are necessary before a converged response is found. Here, it is assumed that $a=b=0.8\;\mathrm{m}, L=0.02\;\mathrm{m}, {h}_{\mathrm{m}}=0.0005\;\mathrm{m},{h}_{\mathrm{c}}=0.001\;\mathrm{m}, \beta ={45}^{^\circ },(0/90/90/0),V=0\;\mathrm{V},{\psi }_{0}=0\;\mathrm{A},{k}_{W}=0\;\mathrm{N}/{\mathrm{m}}^{3}, {c}_{d}=0\;\mathrm{Ns}/{\mathrm{m}}^{3}, \Delta T=0\;\mathrm{K}.$

### Verification study

As the first comparison study, by eliminating plane sound wave, MEE layers, thermal environment, and viscoelastic medium, the first dimensionless natural frequency of a simply supported composite plate for two cases of lay-up orientations are calculated based on the current formulations and the results are compared with those of Ref.^53^ in Table 2.

**Table 2:** Comparison study of non-dimensional natural frequency of a composite plate.

$a/h$	Lay-up			
	$({0}^{^\circ }/{90}^{^\circ })$		$({0}^{^\circ }/{90}^{^\circ }/{90}^{^\circ }/{0}^{^\circ })$	
Study	Ref.^50^	Present	Ref.^50^	Present
20	8.558	8.715	12.277	12.401
100	8.568	8.659	12.277	12.389

In another comparison investigation, by ignoring composite core layer, thermal environment, and viscoelastic medium, the first dimensionless natural frequency (${\widetilde{\omega }}_{11}={\omega }_{11}{a}^{2}\sqrt{{\rho }_{\mathrm{m}}/{c}_{11}}$) of a MEE plate against the different aspect ratios are calculated based on the current formulation and listed in Table 3, which are then compared with those of Ref.^54,55^.

**Table 3:** Comparison study of non-dimensional natural frequency of a MEE plate.

Aspect ratio (a/b)	Present (FSDT)	Ref.^51^ (HSDT)	Ref.^52^ (CPT)
0.5	0.341	0.343	0.366
1	0.532	0.535	0.585
2	1.232	1.233	1.463

**Figure 3:**
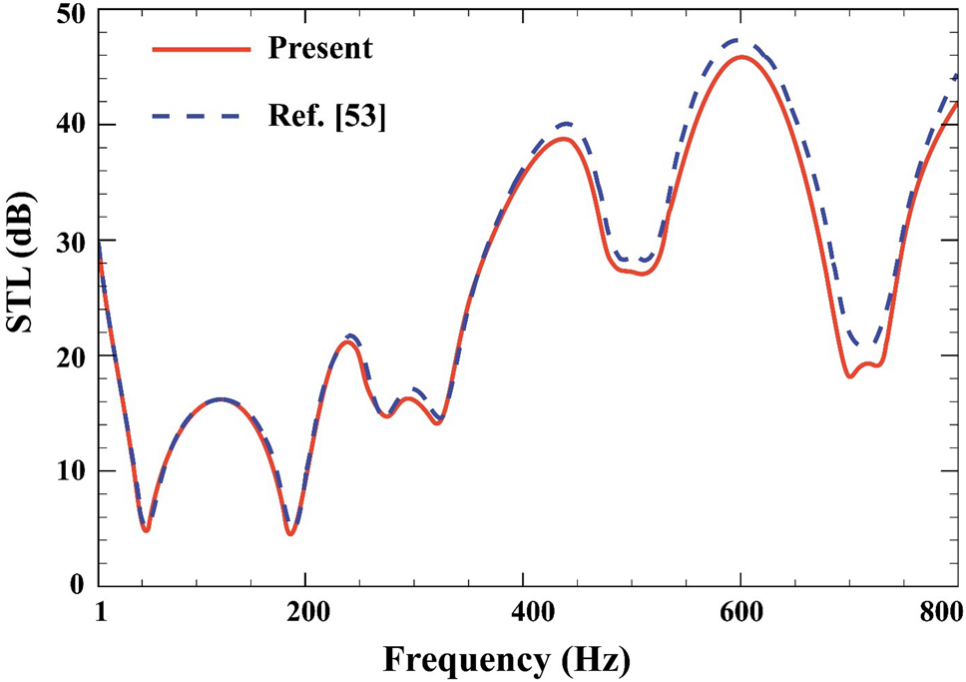
Comparison of STL through the double-walled elastic plate.

Finally, by removing MEE layers, thermal environment, viscoelastic medium, STL across double-walled elastic plate is predicted based on the FSDT for the normal incident sound ($\beta ={0}^{^\circ }$) and is compared with those of Ref.^56^ in Fig. 3 when $E=70\;\text{GPa}$, $\rho =2700 \;\mathrm{kg}/{\mathrm{m}}^{3} ,\; \vartheta =0.3, a=b=0.3\; \mathrm{m}, L=80\; \mathrm{mm}, {h}_{\mathrm{c}}=1\; \mathrm{mm}$. It is observed that the results are close together and are in good agreement.

### Benchmark results

A number of parameters are studied here to observe whether they have a major effect on the value of STL after a sound wave passes through the double-walled composite MEE plate. These parameters are initial electric and magnetic potentials, viscoelastic medium, variations of temperature, ply angle, cavity size and elevation angle of sound wave.

**Figure 4:**
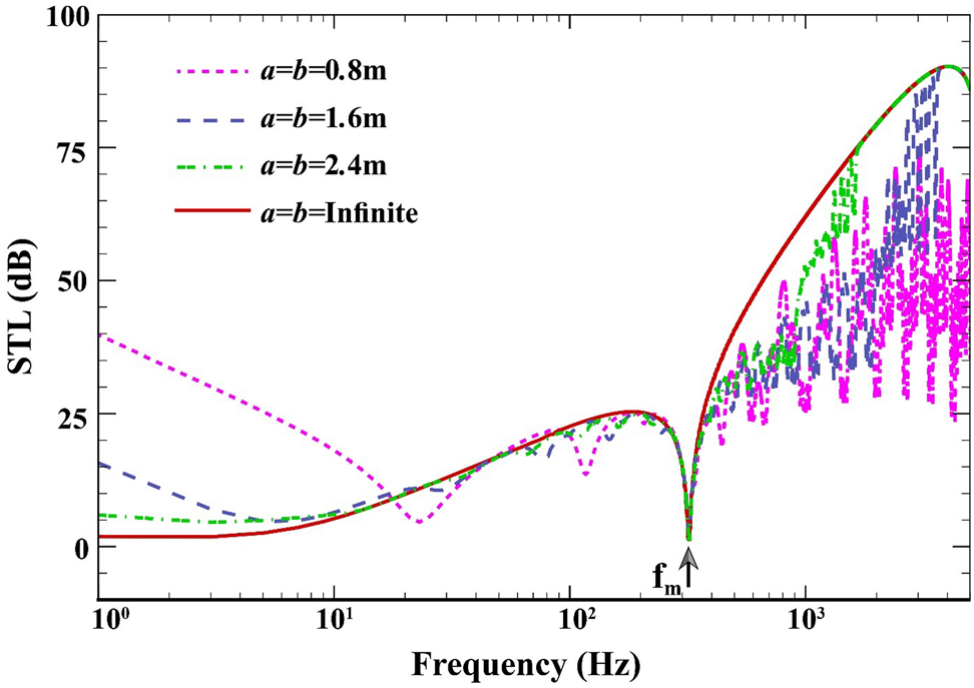
STL of double-walled sandwich composite MEE plate against plate dimensions.

In Fig. 4, the STL through the double-walled sandwich composite MEE plate versus the frequency interval ($1\;\mathrm{Hz}\le f\le 5000\;\text{Hz}$) is presented when $L=0.02\;\mathrm{m}, {h}_{\mathrm{m}}=0.0005\;\mathrm{m},{h}_{\mathrm{c}}=0.001\;\mathrm{m}, \beta ={45}^{^\circ },(0/90/90/0),V=0\;\mathrm{V},{\psi }_{0}=0\;\mathrm{A},{k}_{W}=0\;\mathrm{N}/{\mathrm{m}}^{3}, {c}_{d}=0\;\mathrm{Ns}/{\mathrm{m}}^{3}, \Delta T=0\;\mathrm{K}.$ The mass-air-mass resonance as depicted in the dip of Fig. 4 is a special feature of double-panel systems, and is predicted by^42^

23 $${f}_{\mathrm{m}}=\left(1/2\pi cos\beta \right){\left\{({\rho }_{0} {{c}_{0}}^{2} [{\left({I}_{0} \right)}_{plate1}+{\left({I}_{0}\right)}_{plae2}])/L[{\left({I}_{0}\right)}_{plate1}\times {\left({I}_{0} \right)}_{plate2} ] \right\}}^{1/2}.$$

The required condition for such a phenomenon is the resonance of the two sandwich composite MEE plates over the stiffness of the central air layer. This in turn creates an interval where STL is unsatisfactory. The dips observed before the mass-air-mass resonance are actually the natural frequencies of the overall system. For a larger plate, a smoother STL response whose dips and peaks are kept to a minimum is formed. For the double-walled plate of infinite dimensions, an upper bound for the finite-size partitions is generated after the resonance dip associated with the mass-air-mass dip. This is because the mode-dominated STL is not present in this certain scenario. Over the frequencies smaller than the mass-air-mass resonance, the infinite plate performs better than the finite system in terms of STL response due to the impact of boundary constraints.

**Figure 5:**
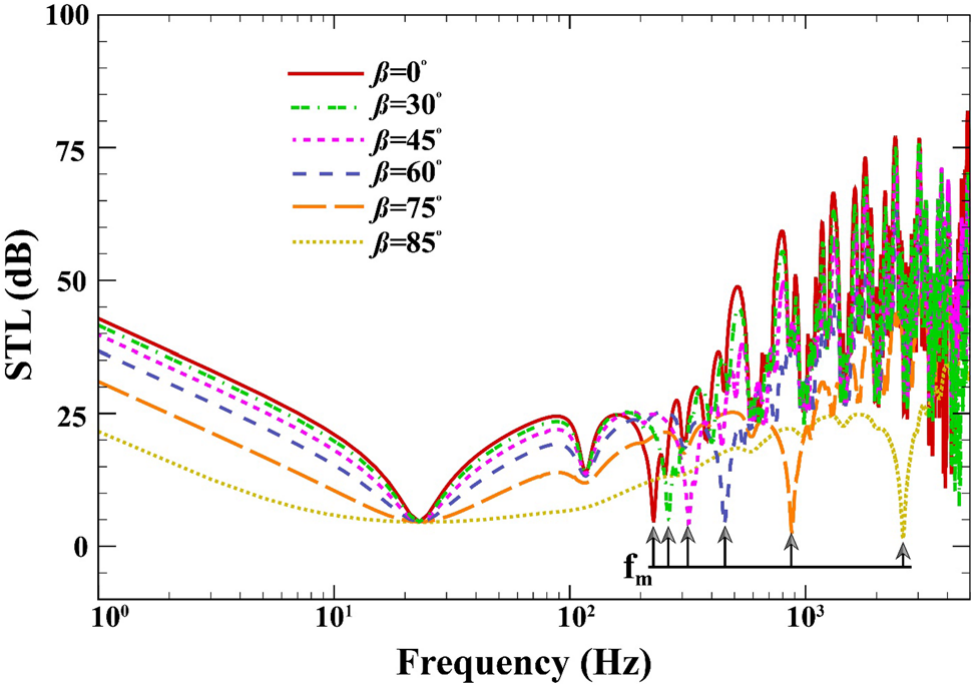
STL of double-walled sandwich composite MEE plate versus the elevation angle.

Figure 5 shows the effect of the elevation angle on the changes of STL across the double-walled sandwich composite MEE plate when $a=b=0.8\;\mathrm{m}, L=0.02\;\mathrm{m}, {h}_{\mathrm{m}}=0.0005\;\mathrm{m},\;{h}_{\mathrm{c}}=0.001\;\mathrm{m}, (0/90/90/0),V=0\;\mathrm{V},{\psi }_{0}=0\;\mathrm{A},{k}_{W}=0\;\mathrm{N}/{\mathrm{m}}^{3}, {c}_{d}=0\;\mathrm{Ns}/{\mathrm{m}}^{3}, \Delta T=0\;\mathrm{K}.$ As observed, there is a direct relationship between the mass-air-mass resonance and the elevation angle. When the elevation angle is decreased, the STL increases, hence improving the noise cancellation behavior of the structure, and vice versa. Before the mass-air-mass resonance, the dips and elevation angle remain independent of one another, and therefore, the plate mode is also independent of the elevation angle of incoming sound wave.

**Figure 6:**
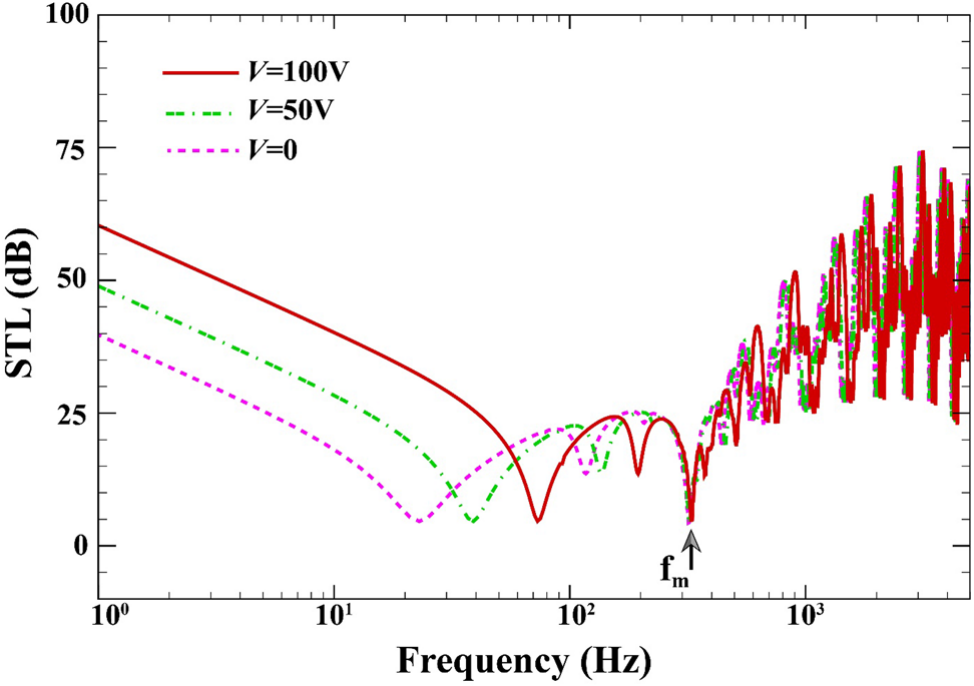
STL of double-walled sandwich composite MEE plate versus the electric voltage.

The STL curves of the double-walled structure in terms of the electric voltage for $a=b=0.8\;\mathrm{m}, L=0.02\;\mathrm{m}, {h}_{\mathrm{m}}=0.0005\;\mathrm{m},{h}_{\mathrm{c}}=0.001\;\mathrm{m}, \beta ={45}^{^\circ },(0/90/90/0),{\psi }_{0}=0\;\mathrm{A},{k}_{W}=0\;\mathrm{N}/{\mathrm{m}}^{3}, {c}_{d}=0\;\mathrm{Ns}/{\mathrm{m}}^{3}, \Delta T=0\;\mathrm{K}$ is displayed in Fig. 6. The soundproofing capability of the developed system can be ameliorated by 20 dB with raising the external electric voltage from 0 to $100\;\mathrm{V}$ (see Refs.^57,58^ for given values), especially over the low-frequency values of mass-air-mass resonance. Expectedly, the amount of electric potential has no impact on the position of mass-air-mass resonance. Furthermore, the mass-air-mass resonance frequency is slightly shifted depending on the external electric voltage. This behavior may be due to the fact that by applying electric voltage to the MEE layers, tensile in-plane and compressive forces created. As a result, this effect can change the vibroacoustic coupling between the two-sandwich magneto-electro-elastic cross-ply layered plate.

**Figure 7:**
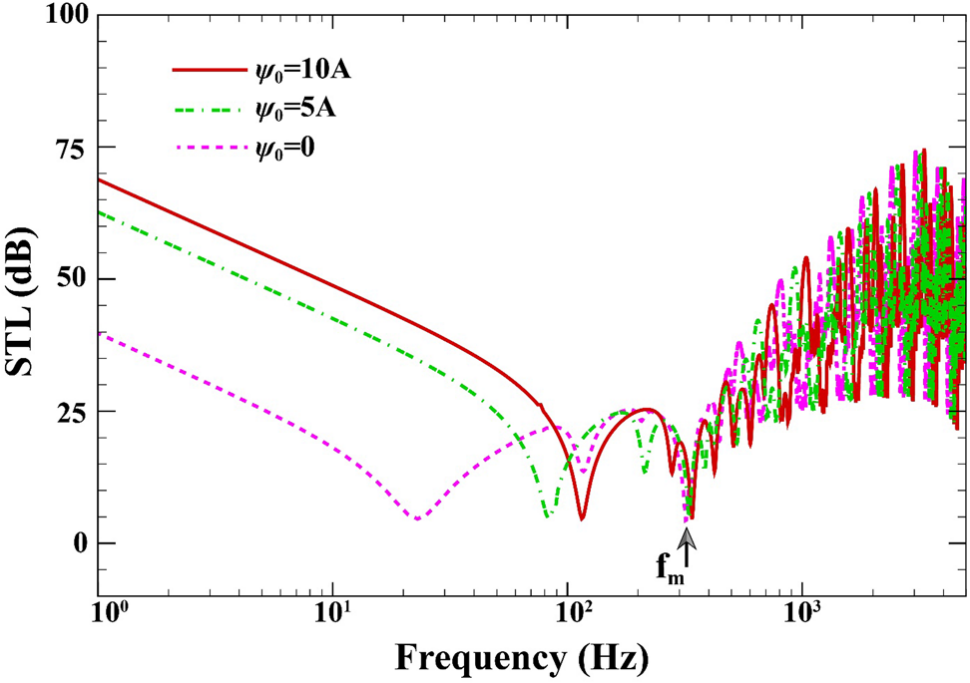
STL of double-walled sandwich composite MEE plate against the external magnetic potential.

Figure 7 indicates the influence of the initial magnetic potential on STL of the double-walled structure when $a=b=0.8\;\mathrm{m}, L=0.02\;\mathrm{m}, {h}_{\mathrm{m}}=0.0005\;\mathrm{m},{h}_{\mathrm{c}}=0.001\;\mathrm{m}, \beta ={45}^{^\circ },(0/90/90/0),V=0\;\mathrm{V},{k}_{W}=0\;\mathrm{N}/{\mathrm{m}}^{3}, {c}_{d}=0\;\mathrm{Ns}/{\mathrm{m}}^{3}, \Delta T=0\;\mathrm{K}.$ The performance of sound transmission loss curves can be enhanced by 29 dB by applying a higher external magnetic potential from 0 to 10A (see Refs.^57,58^ for given values). This is particularly true prior to the mass-air-mass resonance and for the low-frequency values of mass-air-mass resonance. Once again, the position of mass-air-mass resonance remains unrelated to the initial magnetic potential. However, in general, the external magnetic potential seems to be a more important player in the efficacy of sound transmission loss than the electric voltage. Additionally, similar to the effect of electric voltage, the mass-air-mass resonance frequency is slightly shifted depending on the external magnetic potential. This performance may be due to the fact that by exerting the magnetic potential to the MEE layers, tensile in-plane and compressive forces created. Consequently, this effect can change the vibroacoustic coupling between the two-sandwich magneto-electro-elastic cross-ply layered plate.

**Figure 8:**
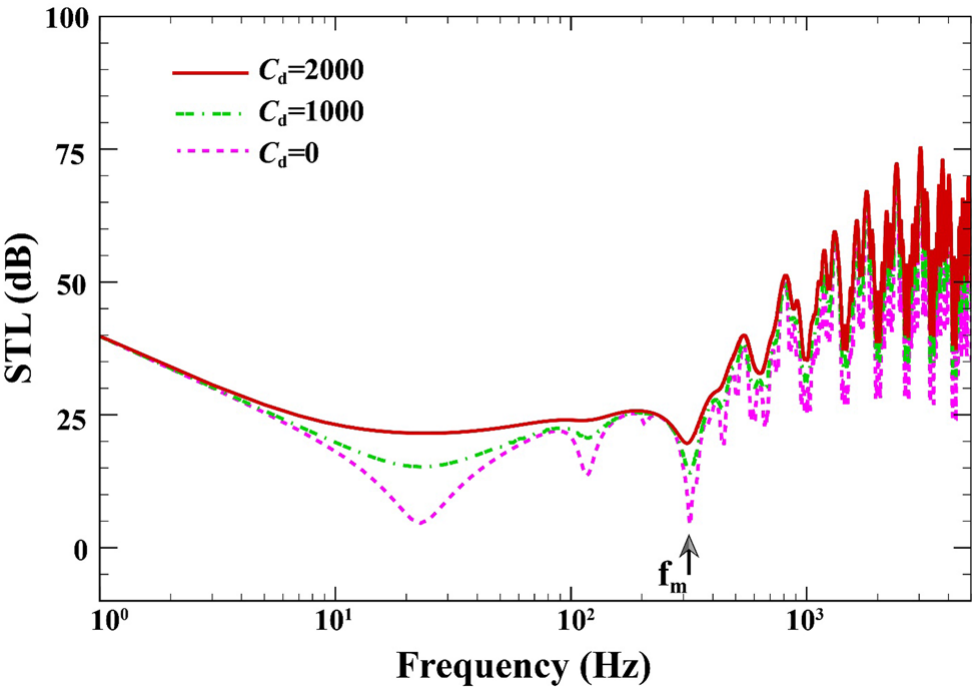
STL of double-walled sandwich composite MEE plate against the viscoelastic medium's damping coefficient.

Figure 8 indicates the variations of STL behavior of the double-walled structure against the different values of the viscoelastic medium's damping coefficient when $a=b=0.8\;\mathrm{m}, L=0.02\;\mathrm{m}, {h}_{\mathrm{m}}=0.0005\;\mathrm{m},{h}_{\mathrm{c}}=0.001\;\mathrm{m}, \beta ={45}^{^\circ },(0/90/90/0),V=0\;\mathrm{V},{\psi }_{0}=0\;\mathrm{A},{k}_{W}=0\;\mathrm{N}/{\mathrm{m}}^{3}, \Delta T=0\;\mathrm{K}.$ It is observed from this figure that by considering damping coefficient from $0\;\mathrm{Ns}/{\mathrm{m}}^{3}$ to 2000 $\mathrm{Ns}/{\mathrm{m}}^{3}$ (see Refs.^59,60^ for given values), STL significantly increases in the whole frequency range, expressly at the mass-air-mass resonance, it increases by 16 dB.

**Figure 9:**
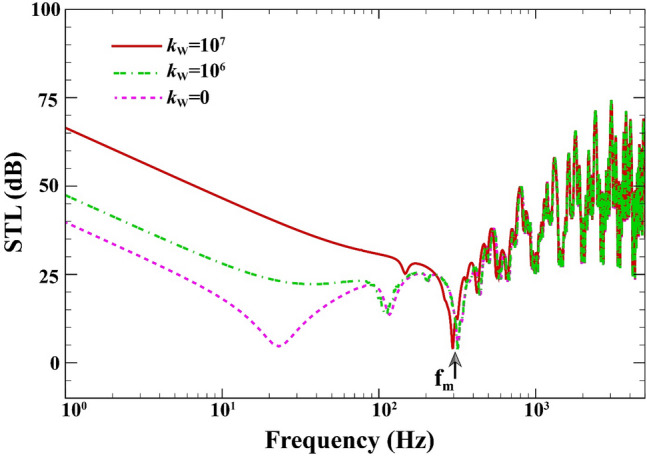
STL of double-walled sandwich composite MEE plate against the viscoelastic medium's stiffness.

Figure 9 displays the effect of the viscoelastic medium's stiffness on the variations of STL curves when $a=b=0.8\;\mathrm{m}, L=0.02\;\mathrm{m}, {h}_{\mathrm{m}}=0.0005\;\mathrm{m},{h}_{\mathrm{c}}=0.001\;\mathrm{m}, \beta ={45}^{^\circ },(0/90/90/0),V=0\;\mathrm{V},{\psi }_{0}=0\;\mathrm{A}, {c}_{d}=0\;\mathrm{Ns}/{\mathrm{m}}^{3}, \Delta T=0\;\mathrm{K}.$ It can be inferred that from this figure that with increasing viscoelastic medium's stiffness coefficient from $0\;\mathrm{N}/{\mathrm{m}}^{3}$ to ${10}^{7}\;\mathrm{N}/{\mathrm{m}}^{3}$(see Refs.^59,60^ for given values), STL due to the increase in the stiffness of the structure (stiffness-hardening effect) increases by 27 dB before the mass-air-mass resonance. However, it is observed that the effect of the viscoelastic medium's stiffness on STL is less in the frequency range above the mass-air-mass resonance.

**Figure 10:**
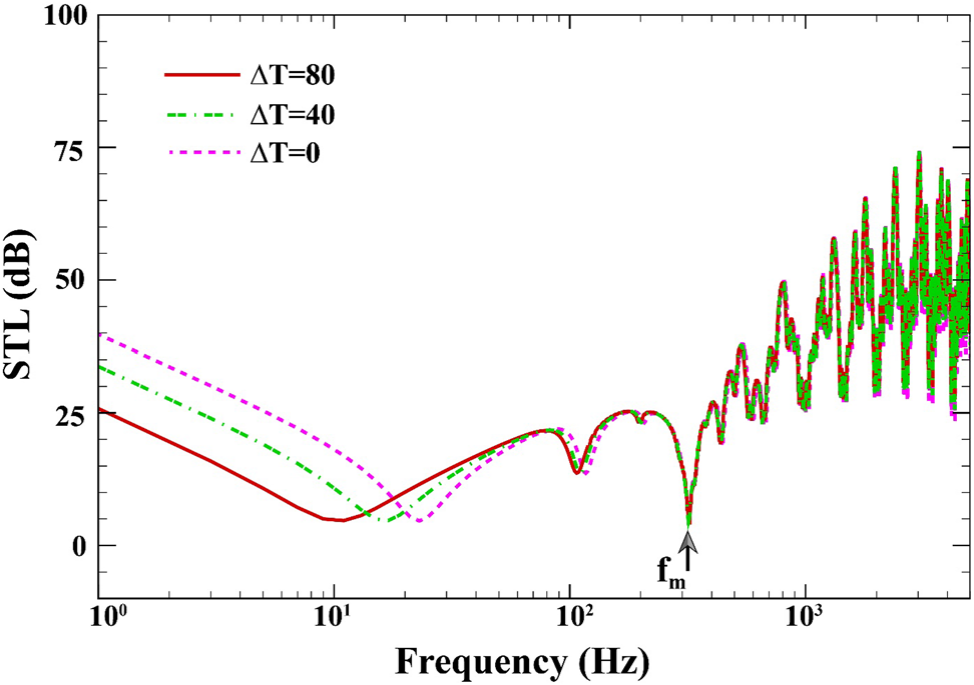
STL of double-walled sandwich composite MEE plate against the temperature rise.

Depicted in Fig. 10 is the effect of temperature rise on the STL curves of sandwich composite MEE plate when $a=b=0.8\;\mathrm{m}, L=0.02\;\mathrm{m}, {h}_{\mathrm{m}}=0.0005\;\mathrm{m},{h}_{\mathrm{c}}=0.001\;\mathrm{m}, \beta ={45}^{^\circ },(0/90/90/0),V=0\;\mathrm{V},{\psi }_{0}=0\;\mathrm{A},{k}_{W}=0\;\mathrm{N}/{\mathrm{m}}^{3}, {c}_{d}=0\;\mathrm{Ns}/{\mathrm{m}}^{3}$. As can be seen in this figure, by increasing the temperature from 0 to $80\;\mathrm{K}$ (see Refs.^61,62^ for given values), the STL decreases by 14 dB before the mass-air-mass resonance. This is because that the temperature changes have a softening effect on the total stiffens of the structure (see Ref.^63^ for more detail).

**Figure 11:**
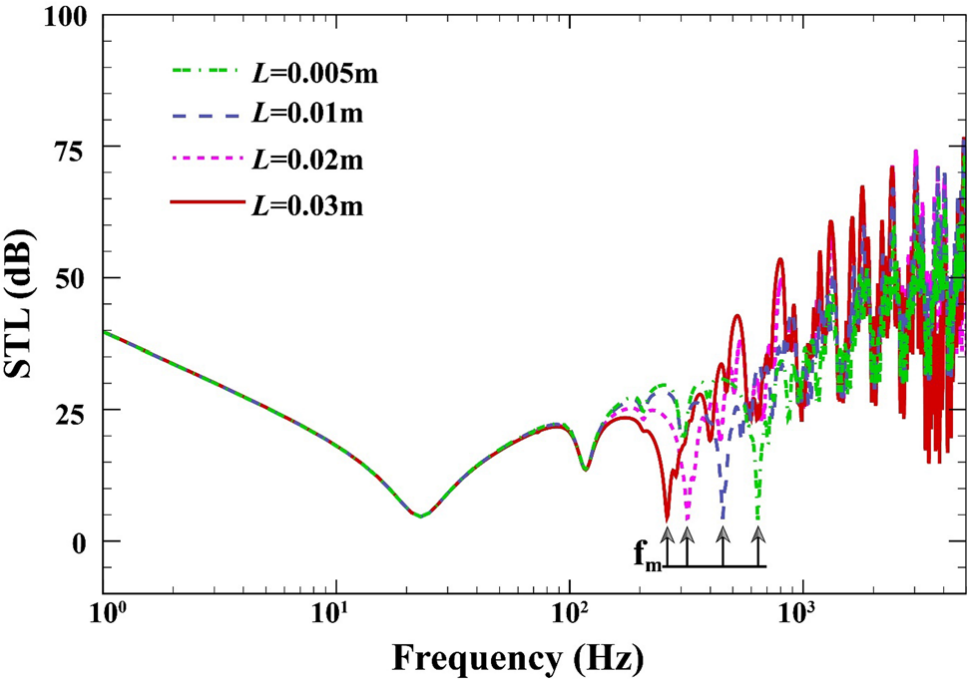
STL of double-walled sandwich composite MEE plate against the air cavity depth.

Figure 11 exhibits the effect of air cavity depth on the STL across of the double-walled sandwich composite MEE plate when $a=b=0.8\;\mathrm{m}, {h}_{\mathrm{m}}=0.0005\;\mathrm{m},{h}_{\mathrm{c}}=0.001\;\mathrm{m}, \beta ={45}^{^\circ },(0/90/90/0),V=0\;\mathrm{V},{\psi }_{0}=0\;\mathrm{A},{k}_{W}=0\;\mathrm{N}/{\mathrm{m}}^{3}, {c}_{d}=0\;\mathrm{Ns}/{\mathrm{m}}^{3}, \Delta T=0\;\mathrm{K}.$ Before the mass-air-mass resonance dip, the existence of acoustic cavity has no impact on the double plate resonance. However, STL response in terms of frequency is considerably altered when the cavity’s depth increases. If the frequency is higher than the mass-air-resonance, a higher depth is associated with a greater STL. In addition, the mass-air-mass resonance dips shift downwards with increasing cavity’s depth as the cavity stiffness decreases in this scenario.

**Figure 12:**
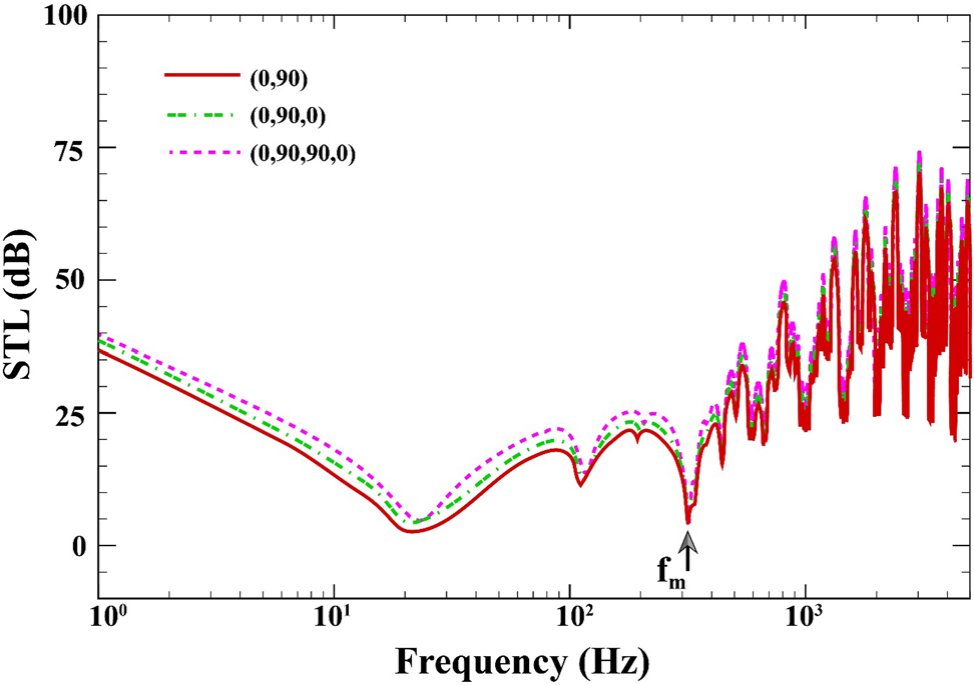
STL of double-walled sandwich composite MEE plate against the laminate layup.

Figure 12 describes the effect of the laminate layup on the changes of STL across the double-walled sandwich composite MEE plate when $a=b=0.8\;\mathrm{m}, L=0.02\;\mathrm{m}, {h}_{\mathrm{m}}=0.0005\;\mathrm{m},{h}_{\mathrm{c}}=0.001\;\mathrm{m}, \beta ={45}^{^\circ }, V=0\;\mathrm{V},{\psi }_{0}=0\;\mathrm{A},{k}_{W}=0\;\mathrm{N}/{\mathrm{m}}^{3}, {c}_{d}=0\;\mathrm{Ns}/{\mathrm{m}}^{3}, \Delta T=0\;\mathrm{K}$. It is clearly seen from this figure that at the low-frequency band, laminate layup $(0/90/90/0)$ increases the STL by 3.5 dB more than the other two cases before the mass-air-mass resonance dip. It is due to the fact this case has a higher bending stiffness and causes the first dip (natural frequency) to shift upwards (see Ref.^52^ for more detail). Furthermore, it is seen that the “mass-air-mass” resonance is independent of the laminate layup.

## Conclusions

This article investigated the parameters affecting the sound transmission behavior of a double-walled composite MEE rectangular plate filled with air resting on the viscoelastic medium in thermal environment. After applying initial magnetic and electric potentials to the structure, the FSDT is employed to model the mechanical response after applying the Hamilton’s principle and considering the normal velocity of fluid and solid on the shared boundaries. In addition to the inspection of transmission loss over the entire studied frequency range, particular attention was paid to the region of mass-air-mass resonance. Some of the most notable findings of this study are mentioned here:

- At the low-frequency band, laminate layup $(0/90/90/0)$ increases the STL more than the other cases.
- The viscoelastic medium improves the STL, particularly, considering damping coefficient, STL significantly increases at the mass-air-mass resonance.
- By increasing the temperature, the STL curve tends to lower frequencies. This behavior is due to the fact that the natural frequencies decrease with increasing temperature changes.
- To improve the acoustic insulation performance, particularly before the mass-air-mass resonance, greater electric and magnetic potentials can be utilized.

## Supplementary Information

Supplementary Information.

## Data Availability

The data and materials in this paper are available on request made directly to the corresponding author.
