# Supplementary material for: Sound transmission loss of double-walled sandwich cross-ply layered magneto-electro-elastic plates under thermal environment
Source: Sci Rep. 2022 Oct 5;12:16621. doi: 10.1038/s41598-022-20965-3 (PMC9534910; doi:10.1038/s41598-022-20965-3)
Supplement: Supplementary file 1 — Supplementary Information. [file 41598_2022_20965_MOESM1_ESM.docx]

**APPENDIX A**

| $\delta u_{i}: a_{11}\frac{\partial^{2}u_{i}}{\partial x^{2}}+a_{12}\frac{\partial^{2}v_{i}}{\partial x\partial y}+b_{11}\frac{\partial^{2}\theta_{xi}}{\partial x^{2}}+b_{12}\frac{\partial^{2}\theta_{yi}}{\partial x\partial y}+a_{66}\left( \frac{\partial^{2}u_{i}}{\partial y^{2}}+\frac{\partial^{2}v_{i}}{\partial x\partial y} \right)+b_{66}\left( \frac{\partial^{2}\theta_{xi}}{\partial y^{2}}+\frac{\partial^{2}\theta_{yi}}{\partial x\partial y} \right)+g_{11}\frac{\partial\bar{\Upsilon}_{i}}{\partial x}+l_{11}\frac{\partial\bar{\Psi}_{i}}{\partial x}=I_{0}\frac{\partial^{2}u_{i}}{\partial t^{2}}+I_{1}\frac{\partial^{2}\theta_{xi}}{\partial t^{2}},$  $\delta v_{i}: a_{66}\left( \frac{\partial^{2}v_{i}}{\partial x^{2}}+\frac{\partial^{2}u_{i}}{\partial x\partial y} \right)+b_{66}\left( \frac{\partial^{2}\theta_{xi}}{\partial x\partial y}+\frac{\partial^{2}\theta_{yi}}{\partial x^{2}} \right)+a_{12}\frac{\partial^{2}u_{i}}{\partial x\partial y}+a_{22}\frac{\partial^{2}v_{i}}{\partial y^{2}}+b_{12}\frac{\partial^{2}\theta_{xi}}{\partial x\partial y}+b_{22}\frac{\partial^{2}\theta_{yi}}{\partial y^{2}}+g_{11}\frac{\partial\bar{\Upsilon}_{i}}{\partial y}+l_{11}\frac{\partial\bar{\Psi}_{i}}{\partial y}=I_{0}\frac{\partial^{2}v_{i}}{\partial t^{2}}+I_{1}\frac{\partial^{2}\theta_{yi}}{\partial t^{2}},$  $\delta w_{i}: k_{s}a_{55}\left( \frac{\partial\theta_{xi}}{\partial x}+\frac{\partial^{2}w_{i}}{\partial x^{2}} \right)+k_{s}a_{44}\left( \frac{\partial\theta_{yi}}{\partial y}+\frac{\partial^{2}w_{i}}{\partial y^{2}} \right)+\left( N_{x}^{E}+N_{x}^{M}+N_{x}^{T} \right)\frac{\partial^{2}w_{i}}{\partial x^{2}}+\left( N_{y}^{E}+N_{y}^{M}+N_{y}^{T} \right)\frac{\partial^{2}w_{i}}{\partial y^{2}}+k_{W}w_{2}+c_{d}\frac{\partial w_{2}}{\partial t}-k_{s}r_{2}\left( \frac{\partial^{2}\bar{\Upsilon}_{i}}{\partial x^{2}}+\frac{\partial^{2}\bar{\Upsilon}_{i}}{\partial y^{2}} \right)-k_{s}r_{3}\left( \frac{\partial^{2}\bar{\Psi}_{i}}{\partial x^{2}}+\frac{\partial^{2}\bar{\Psi}_{i}}{\partial y^{2}} \right)=I_{0}\frac{\partial^{2}w_{i}}{\partial t^{2}}+q_{i},$  $\delta\theta_{xi}: b_{11}\frac{\partial^{2}u_{i}}{\partial x^{2}}+b_{12}\frac{\partial^{2}v_{i}}{\partial x\partial y}+d_{11}\frac{\partial^{2}\theta_{xi}}{\partial x^{2}}+d_{12}\frac{\partial^{2}\theta_{yi}}{\partial x\partial y}+b_{66}\left( \frac{\partial^{2}u_{i}}{\partial y^{2}}+\frac{\partial^{2}v_{i}}{\partial x\partial y} \right)+d_{66}\left( \frac{\partial^{2}\theta_{xi}}{\partial y^{2}}+\frac{\partial^{2}\theta_{yi}}{\partial x\partial y} \right)-k_{s}a_{55}\left( \theta_{xi}+\frac{\partial w_{i}}{\partial x} \right)+\left( g_{22}+k_{s}r_{2} \right)\frac{\partial\bar{\Upsilon}_{i}}{\partial x}+\left( l_{22}+k_{s}r_{3} \right)\frac{\partial\bar{\Psi}_{i}}{\partial x}=I_{1}\frac{\partial^{2}u_{i}}{\partial t^{2}}+I_{2}\frac{\partial^{2}\theta_{xi}}{\partial t^{2}},$  $\delta\theta_{yi}: b_{12}\frac{\partial^{2}u_{i}}{\partial x\partial y}+b_{22}\frac{\partial^{2}v_{i}}{\partial y^{2}}+d_{12}\frac{\partial^{2}\theta_{xi}}{\partial x\partial y}+d_{22}\frac{\partial^{2}\theta_{yi}}{\partial y^{2}}+b_{66}\left( \frac{\partial^{2}u_{i}}{\partial x\partial y}+\frac{\partial^{2}v_{i}}{\partial x^{2}} \right)+d_{66}\left( \frac{\partial^{2}\theta_{xi}}{\partial x\partial y}+\frac{\partial^{2}\theta_{yi}}{\partial x^{2}} \right)-k_{s}a_{44}\left( \theta_{yi}+\frac{\partial w_{i}}{\partial y} \right)+\left( g_{22}+k_{s}r_{2} \right)\frac{\partial\bar{\Upsilon}_{i}}{\partial y}+\left( l_{22}+k_{s}r_{3} \right)\frac{\partial\bar{\Psi}_{i}}{\partial y}=I_{1}\frac{\partial^{2}v_{i}}{\partial t^{2}}+I_{2}\frac{\partial^{2}\theta_{yi}}{\partial t^{2}}$,  $\delta\bar{\Upsilon}_{i}: q_{1}\left( \frac{\partial^{2}\bar{\Upsilon}_{i}}{\partial x^{2}}+\frac{\partial^{2}\bar{\Upsilon}_{i}}{\partial y^{2}} \right)-p_{1}\bar{\Upsilon}_{i}+q_{2}\left( \frac{\partial^{2}\bar{\Psi}_{i}}{\partial x^{2}}+\frac{\partial^{2}\bar{\Psi}_{i}}{\partial y^{2}} \right)-p_{2}\bar{\Psi}_{i}+k_{s} r_{2}\left( \frac{\partial^{2}w_{i}}{\partial y^{2}}+\frac{\partial^{2}w_{i}}{\partial x^{2}} \right)+g_{11}\left( \frac{\partial u_{i}}{\partial x}+\frac{\partial v_{i}}{\partial y} \right)+\left( g_{22}+k_{s}r_{2} \right)\left( \frac{\partial\theta_{xi}}{\partial x}+\frac{\partial\theta_{yi}}{\partial y} \right)=0,$  $\delta\bar{\Psi}_{i}:q_{2}\left( \frac{\partial^{2}\bar{\Upsilon}_{i}}{\partial x^{2}}+\frac{\partial^{2}\bar{\Upsilon}_{i}}{\partial y^{2}} \right)-p_{2}\bar{\Upsilon}_{i}+q_{3}\left( \frac{\partial^{2}\bar{\Psi}_{i}}{\partial x^{2}}+\frac{\partial^{2}\bar{\Psi}_{i}}{\partial y^{2}} \right)-p_{3}\bar{\Psi}_{i}+k_{s}r_{3}\left( \frac{\partial^{2}w_{i}}{\partial y^{2}}+\frac{\partial^{2}w_{i}}{\partial x^{2}} \right)+l_{11}\left( \frac{\partial u_{i}}{\partial x}+\frac{\partial v_{i}}{\partial y} \right)+\left( l_{22}+k_{s}r_{3} \right)\left( \frac{\partial\theta_{xi}}{\partial x}+\frac{\partial\theta_{yi}}{\partial y} \right)=0,$ | (A1)  (A2)  (A3)  (A4)  (A5)  (A6)  (A7) |
| --- | --- |

where

$a_{11}=\int_{-h_{m}-h_{c}/2}^{-h_{c}/2} c_{11}dz+\sum_{k=1}^{N} \bar{Q}_{11}\left( h_{k}-h_{k-1} \right)+\int_{h_{c}/2}^{h_{c}/2+h_{m}} c_{11}dz$ ,

$b_{11}=\int_{-h_{m}-h_{c}/2}^{-h_{c}/2} c_{11}\mathrm{zd}z+\frac{1}{2}\sum_{k=1}^{N} \bar{Q}_{11}\left( {h_{k}}^{2}-{h_{k-1}}^{2} \right)+\int_{h_{c}/2}^{h_{c}/2+h_{m}} c_{11}\mathrm{zd}z$ ,

$d_{11}=\int_{-h_{m}-h_{c}/2}^{-h_{c}/2} c_{11}z^{2}dz+\frac{1}{3}\sum_{k=1}^{N} \bar{Q}_{11}\left( {h_{k}}^{3}-{h_{k-1}}^{3} \right)+\int_{h_{c}/2}^{h_{c}/2+h_{m}} c_{11}z^{2}dz$ ,

$a_{12}=\int_{-h_{m}-h_{c}/2}^{-h_{c}/2} c_{12}dz+\sum_{k=1}^{N} \bar{Q}_{12}\left( h_{k}-h_{k-1} \right)+\int_{h_{c}/2}^{h_{c}/2+h_{m}} c_{12}dz$ ,

$b_{12}=\int_{-h_{m}-h_{c}/2}^{-h_{c}/2} c_{12}\mathrm{zd}z+\frac{1}{2}\sum_{k=1}^{N} \bar{Q}_{12}\left( {h_{k}}^{2}-{h_{k-1}}^{2} \right)+\int_{h_{c}/2}^{h_{c}/2+h_{m}} c_{12}\mathrm{zd}z$ ,

$d_{12}=\int_{-h_{m}-h_{c}/2}^{-h_{c}/2} c_{12}z^{2}dz+\frac{1}{3}\sum_{k=1}^{N} \bar{Q}_{12}\left( {h_{k}}^{3}-{h_{k-1}}^{3} \right)+\int_{h_{c}/2}^{h_{c}/2+h_{m}} c_{12}z^{2}dz$ ,

$a_{22}=\int_{-h_{m}-h_{c}/2}^{-h_{c}/2} c_{22}dz+\sum_{k=1}^{N} \bar{Q}_{22}\left( h_{k}-h_{k-1} \right)+\int_{h_{c}/2}^{h_{c}/2+h_{m}} c_{22}dz$ ,

$b_{22}=\int_{-h_{m}-h_{c}/2}^{-h_{c}/2} c_{22}\mathrm{zd}z+\frac{1}{2}\sum_{k=1}^{N} \bar{Q}_{22}\left( {h_{k}}^{2}-{h_{k-1}}^{2} \right)+\int_{h_{c}/2}^{h_{c}/2+h_{m}} c_{22}\mathrm{zd}z$ ,

$d_{22}=\int_{-h_{m}-h_{c}/2}^{-h_{c}/2} c_{22}z^{2}dz+\frac{1}{3}\sum_{k=1}^{N} \bar{Q}_{22}\left( {h_{k}}^{3}-{h_{k-1}}^{3} \right)+\int_{h_{c}/2}^{h_{c}/2+h_{m}} c_{22}z^{2}dz$ ,

$a_{66}=\int_{-h_{m}-h_{c}/2}^{-h_{c}/2} c_{66}dz+\sum_{k=1}^{N} \bar{Q}_{11}\left( h_{k}-h_{k-1} \right)+\int_{h_{c}/2}^{h_{c}/2+h_{m}} c_{11}dz$ ,

$b_{66}=\int_{-h_{m}-h_{c}/2}^{-h_{c}/2} c_{66}\mathrm{zd}z+\frac{1}{2}\sum_{k=1}^{N} \bar{Q}_{66}\left( {h_{k}}^{2}-{h_{k-1}}^{2} \right)+\int_{h_{c}/2}^{h_{c}/2+h_{m}} c_{66}\mathrm{zd}z$ ,

$d_{66}=\int_{-h_{m}-h_{c}/2}^{-h_{c}/2} c_{66}z^{2}dz+\frac{1}{3}\sum_{k=1}^{N} \bar{Q}_{66}\left( {h_{k}}^{3}-{h_{k-1}}^{3} \right)+\int_{h_{c}/2}^{h_{c}/2+h_{m}} c_{66}z^{2}dz$ ,

$\left\{ g_{11},g_{22} \right\}=\int_{-h_{m}-h_{c}/2}^{-h_{c}/2} e_{31}\left( \frac{\pi}{h_{m}} \right)\sin\left\{ \frac{\pi\left[ +\left( \frac{h_{c}+h_{m}}{2} \right) \right]}{h_{m}} \right\}\left\{ 1,z \right\}dz+\int_{h_{c}/2}^{h_{c}/2+h_{m}} e_{31}\left( \frac{\pi}{h_{m}} \right)\sin\left\{ \frac{\pi\left[ -\left( \frac{h_{c}+h_{m}}{2} \right) \right]}{h_{m}} \right\}\left\{ 1,z \right\}dz,$

$\left\{ l_{11},l_{22} \right\}=\int_{-h_{m}-h_{c}/2}^{-h_{c}/2} f_{31}\left( \frac{\pi}{h_{m}} \right)\sin\left\{ \frac{\pi\left[ +\left( \frac{h_{c}+h_{m}}{2} \right) \right]}{h_{m}} \right\}\left\{ 1,z \right\}dz+\int_{h_{c}/2}^{h_{c}/2+h_{m}} f_{31}\left( \frac{\pi}{h_{m}} \right)\sin\left\{ \frac{\pi\left[ -\left( \frac{h_{c}+h_{m}}{2} \right) \right]}{h_{m}} \right\}\left\{ 1,z \right\}dz,$

$r_{2}=\int_{-h_{m}-h_{c}/2}^{-h_{c}/2} e_{15}\cos\left\{ \frac{\pi\left[ +\left( \frac{h_{c}+h_{m}}{2} \right) \right]}{h_{m}} \right\}dz+\int_{h_{c}/2}^{h_{c}/2+h_{m}} e_{15}\cos\left\{ \frac{\pi\left[ -\left( \frac{h_{c}+h_{m}}{2} \right) \right]}{h_{m}} \right\}dz,$

$r_{3}=\int_{-h_{m}-h_{c}/2}^{-h_{c}/2} f_{15}\cos\left\{ \frac{\pi\left[ +\left( \frac{h_{c}+h_{m}}{2} \right) \right]}{h_{m}} \right\}dz+\int_{h_{c}/2}^{h_{c}/2+h_{m}} f_{15}\cos\left\{ \frac{\pi\left[ -\left( \frac{h_{c}+h_{m}}{2} \right) \right]}{h_{m}} \right\}dz,$

$q_{1}=\int_{-h_{m}-h_{c}/2}^{-h_{c}/2} \kappa_{11}\left[ \cos\left\{ \frac{\pi\left[ +\left( \frac{h_{c}+h_{m}}{2} \right) \right]}{h_{m}} \right\} \right]^{2}dz+\int_{h_{c}/2}^{h_{c}/2+h_{m}} \kappa_{11}\left[ \cos\left\{ \frac{\pi\left[ -\left( \frac{h_{c}+h_{m}}{2} \right) \right]}{h_{m}} \right\} \right]^{2}dz,$

$q_{2}=\int_{-h_{m}-h_{c}/2}^{-h_{c}/2} \mu_{11}\left[ \cos\left\{ \frac{\pi\left[ +\left( \frac{h_{c}+h_{m}}{2} \right) \right]}{h_{m}} \right\} \right]^{2}dz+\int_{h_{c}/2}^{h_{c}/2+h_{m}} \mu_{11}\left[ \cos\left\{ \frac{\pi\left[ -\left( \frac{h_{c}+h_{m}}{2} \right) \right]}{h_{m}} \right\} \right]^{2}dz,$

$p_{1}=\int_{-h_{m}-\frac{h_{c}}{2}}^{-\frac{h_{c}}{2}} \kappa_{33}\left[ \frac{\pi}{h_{m}}\sin\left\{ \frac{\pi\left[ +\left( \frac{h_{c}+h_{m}}{2} \right) \right]}{h_{m}} \right\} \right]^{2}dz+\int_{\frac{h_{c}}{2}}^{\frac{h_{c}}{2}+h_{m}} \kappa_{33}\left[ \frac{\pi}{h_{m}}\sin\left\{ \frac{\pi\left[ -\left( \frac{h_{c}+h_{m}}{2} \right) \right]}{h_{m}} \right\} \right]^{2}dz,$

$p_{2}=\int_{-h_{m}-\frac{h_{c}}{2}}^{-\frac{h_{c}}{2}} \mu_{33}\left[ \frac{\pi}{h_{m}}\sin\left\{ \frac{\pi\left[ +\left( \frac{h_{c}+h_{m}}{2} \right) \right]}{h_{m}} \right\} \right]^{2}dz+\int_{\frac{h_{c}}{2}}^{\frac{h_{c}}{2}+h_{m}} \mu_{33}\left[ \frac{\pi}{h_{m}}\sin\left\{ \frac{\pi\left[ -\left( \frac{h_{c}+h_{m}}{2} \right) \right]}{h_{m}} \right\} \right]^{2}dz,$

$q_{3}=\int_{-h_{m}-h_{c}/2}^{-h_{c}/2} \gamma_{11}\left[ \cos\left\{ \frac{\pi\left[ +\left( \frac{h_{c}+h_{m}}{2} \right) \right]}{h_{m}} \right\} \right]^{2}dz+\int_{h_{c}/2}^{h_{c}/2+h_{m}} \gamma_{11}\left[ \cos\left\{ \frac{\pi\left[ -\left( \frac{h_{c}+h_{m}}{2} \right) \right]}{h_{m}} \right\} \right]^{2}dz,$

$p_{3}=\int_{-h_{m}-\frac{h_{c}}{2}}^{-\frac{h_{c}}{2}} \gamma_{33}\left[ \frac{\pi}{h_{m}}\sin\left\{ \frac{\pi\left[ +\left( \frac{h_{c}+h_{m}}{2} \right) \right]}{h_{m}} \right\} \right]^{2}dz+\int_{\frac{h_{c}}{2}}^{\frac{h_{c}}{2}+h_{m}} \gamma_{33}\left[ \frac{\pi}{h_{m}}\sin\left\{ \frac{\pi\left[ -\left( \frac{h_{c}+h_{m}}{2} \right) \right]}{h_{m}} \right\} \right]^{2}dz,$

**Appendix B**

$\kappa_{1,1}=\kappa_{8,8}=-a_{11}{(m\pi/a)}^{2}-a_{66}{(n\pi/b)}^{2}+I_{0}\omega^{2}$ ,

$\kappa_{1,2}=\kappa_{2,1}=\kappa_{8,9}=\kappa_{9,8}=-\left( a_{12}+a_{66} \right)\left( m\pi/a \right)\left( n\pi/b \right)$,

$\kappa_{1,4}=\kappa_{4,1}=\kappa_{8,11}=\kappa_{11,8}=-b_{11}\left( m\pi/a \right)^{2}-b_{66}\left( n\pi/b \right)^{2}+I_{1}\omega^{2}$ ,

$\kappa_{1,5}=\kappa_{5,1}=\kappa_{8,12}=\kappa_{12,8}=-\left( b_{12}+b_{66} \right)\left( m\pi/a \right)\left( n\pi/b \right)$ ,

$\kappa_{1,6}=-\kappa_{6,1}=\mathcal{L}_{8,13}=-\mathcal{L}_{13,8}=g_{11}\left( m\pi/a \right)$ ,

$\kappa_{1,7}=-\kappa_{7,1}=\kappa_{8,14}=-\kappa_{14,8}=l_{11}\left( n\pi/b \right)$ ,

$\kappa_{2,2}=\kappa_{9,9}=-a_{66}{(m\pi/a)}^{2}-a_{22}\left( n\pi/b \right)^{2}+I_{0}\omega^{2}$ ,

$\kappa_{2,4}=\kappa_{4,2}=\kappa_{9,11}=\kappa_{11,9}=-\left( b_{12}+b_{66} \right)\left( m\pi/a \right)\left( n\pi/b \right)$ ,

$\kappa_{2,5}=\kappa_{5,2}=\kappa_{9,12}=\kappa_{12,9}=-b_{66}\left( m\pi/a \right)^{2}-\left( n\pi/b \right)^{2}+I_{1}\omega^{2}$ ,

$\kappa_{2,6}=-\kappa_{6,2}=\kappa_{9,13}=-\kappa_{13,9}=g_{11}\left( n\pi/b \right)$,

$\kappa_{2,7}=-\kappa_{7,2}=\kappa_{9,14}=-\kappa_{14,9}=l_{11}\left( n\pi/b \right)$,

$\kappa_{3,3}=\kappa_{10,10}=-k_{s}a_{55}\left( \frac{m\pi}{a} \right)^{2}-k_{s}a_{44}\left( \frac{n\pi}{b} \right)^{2}-\left( N_{x}^{E}+N_{x}^{M}+N_{x}^{T} \right)\left( \frac{m\pi}{a} \right)^{2}-\left( N_{y}^{E}+N_{y}^{M}+N_{y}^{T} \right){(n\pi/b)}^{2}+k_{W}+c_{d}j\omega+I_{0}\omega^{2}+2\omega^{2} j\rho_{0} e^{-jk_{z}L}/\left[ k_{z} (e^{-jk_{z}L}-e^{jk_{z}L}) \right]$ ,

$\kappa_{3,4}=\kappa_{4,3}=\kappa_{10,11}=\kappa_{11,10}=-k_{s}a_{55}\left( m\pi/a \right)$,

$\kappa_{3,5}=\kappa_{5,3}=\kappa_{10,12}=\kappa_{12,10}=-k_{s}a_{44}\left( n\pi/b \right)$,

$\kappa_{3,6}=-\kappa_{6,3}=\kappa_{10,13}=-\kappa_{13,10}=k_{s}r_{2}\left[ {(m\pi/a)}^{2}+{(n\pi/b)}^{2} \right]$,

$\kappa_{3,7}=-\kappa_{7,3}=\kappa_{10,14}=-\kappa_{14,10}=k_{s}r_{3}\left[ {(m\pi/a)}^{2}+{(n\pi/b)}^{2} \right]$,

$\kappa_{3,10}=\kappa_{10,3}=-2j\omega^{2}\rho_{0} /\left[ k_{z} (e^{-jk_{z}L}-e^{jk_{z}L}) \right],$

$\kappa_{4,4}=\kappa_{11,11}=-d_{11}\left( m\pi/a \right)^{2}-d_{66}\left( n\pi/b \right)^{2}-k_{s}a_{55}+I_{2}\omega^{2}$,

$\kappa_{4,5}=\kappa_{5,4}=\kappa_{12,11}=\kappa_{1110}=-\left( d_{12}+d_{66} \right)\left( m\pi/a \right)\left( n\pi/b \right)$ ,

$\kappa_{4,6}=-\kappa_{6,4}=\kappa_{11,13}=-\kappa_{13,11}=\left( g_{22}+k_{s}r_{2} \right)\left( m\pi/a \right)$ ,

$\kappa_{4,7}=-\kappa_{7,4}=\kappa_{11,14}=-\kappa_{14,11}=\left( l_{22}+k_{s}r_{3} \right)\left( m\pi/a \right)$ ,

$\kappa_{5,5}=\kappa_{12,12}=d_{22}{(n\pi/b)}^{2}-d_{66}\left( m\pi/a \right)^{2}-k_{s}a_{44}+I_{2}\omega^{2}$

$\kappa_{5,6}=-\kappa_{6,5}=\kappa_{12,13}=-\kappa_{13,12}=\left( g_{22}+k_{s}r_{2} \right)\left( n\pi/b \right)$ ,

$\kappa_{5,7}=-\kappa_{7,5}=\kappa_{12,14}=-\kappa_{14,12}=\left( l_{22}+k_{s}r_{3} \right)\left( n\pi/b \right)$ ,

$\kappa_{6,6}=\kappa_{13,13}=q_{1}\left[ {(m\pi/a)}^{2}+{(n\pi/b)}^{2} \right]-p_{1}$,

$\kappa_{6,7}=\kappa_{7,6}=\kappa_{13,14}=\kappa_{14,13}=q_{2}\left[ {(m\pi/a)}^{2}+{(n\pi/b)}^{2} \right]-p_{2},$

$\kappa_{7,7}=\kappa_{14,14}=q_{3}\left[ {(m\pi/a)}^{2}+{(n\pi/b)}^{2} \right]-p_{3}$

$F=2j\rho_{0}\omega I_{mn}e^{-jk_{z}\left( L+2h_{m}+h_{c} \right)}$,
